# Tumor-suppressive disruption of cancer subtype-associated super enhancer circuits by small molecule treatment

**DOI:** 10.1093/narcan/zcad007

**Published:** 2023-02-06

**Authors:** Anke Koeniger, Pierfrancesco Polo, Anna Brichkina, Florian Finkernagel, Alexander Visekruna, Andrea Nist, Thorsten Stiewe, Michael Daude, Wibke E Diederich, Thomas M Gress, Till Adhikary, Matthias Lauth

**Affiliations:** Philipps University Marburg, Dept. of Gastroenterology, Endocrinology and Metabolism, Center for Tumor- and Immune Biology, 35043 Marburg, Germany; Philipps University Marburg, Dept. of Gastroenterology, Endocrinology and Metabolism, Center for Tumor- and Immune Biology, 35043 Marburg, Germany; Philipps University Marburg, Dept. of Gastroenterology, Endocrinology and Metabolism, Center for Tumor- and Immune Biology, 35043 Marburg, Germany; Philipps University Marburg, Bioinformatics Core Facility, Center for Tumor- and Immune Biology, 35043 Marburg, Germany; Philipps University Marburg, Institute for Medical Microbiology and Hygiene, 35043 Marburg, Germany; Member of the German Center for Lung Research (DZL), Center for Tumor- and Immune Biology, Genomics Core Facility, Institute of Molecular Oncology, Philipps University Marburg, 35043 Marburg, Germany; Member of the German Center for Lung Research (DZL), Center for Tumor- and Immune Biology, Genomics Core Facility, Institute of Molecular Oncology, Philipps University Marburg, 35043 Marburg, Germany; Philipps University Marburg, Core Facility Medical Chemistry, Center for Tumor- and Immune Biology, 35043 Marburg, Germany; Philipps University Marburg, Dept. of Medicinal Chemistry and Core Facility Medical Chemistry, Center for Tumor- and Immune Biology, 35043 Marburg, Germany; Philipps University Marburg, Dept. of Gastroenterology, Endocrinology and Metabolism, Center for Tumor- and Immune Biology, 35043 Marburg, Germany; Philipps University Marburg, Institute for Medical Bioinformatics and Biostatistics and Institute for Molecular Biology and Tumor Research, Marburg, Germany; Philipps University Marburg, Dept. of Gastroenterology, Endocrinology and Metabolism, Center for Tumor- and Immune Biology, 35043 Marburg, Germany

## Abstract

Transcriptional cancer subtypes which correlate with traits such as tumor growth, drug sensitivity or the chances of relapse and metastasis, have been described for several malignancies. The core regulatory circuits (CRCs) defining these subtypes are established by chromatin super enhancers (SEs) driving key transcription factors (TFs) specific for the particular cell state. In neuroblastoma (NB), one of the most frequent solid pediatric cancer entities, two major SE-directed molecular subtypes have been described: A more lineage-committed adrenergic (ADRN) and a mesenchymal (MES) subtype. Here, we found that a small isoxazole molecule (ISX), a frequently used pro-neural drug, reprogrammed SE activity and switched NB cells from an ADRN subtype towards a growth-retarded MES-like state. The MES-like state shared strong transcriptional overlap with ganglioneuroma (GN), a benign and highly differentiated tumor of the neural crest. Mechanistically, ISX suppressed chromatin binding of N-MYC, a CRC-amplifying transcription factor, resulting in loss of key ADRN subtype-enriched components such as N-MYC itself, PHOX2B and ALK, while concomitently, MES subtype markers were induced. Globally, ISX treatment installed a chromatin accessibility landscape typically associated with low risk NB. In summary, we provide evidence that CRCs and cancer subtype reprogramming might be amenable to future therapeutic targeting.

## Graphical Abstract

**Graphical Abstract:**
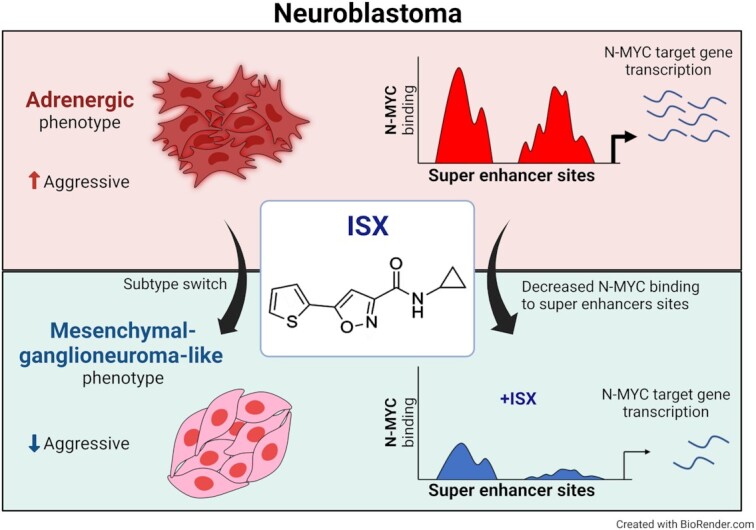
Tumor subtype modulation by the small molecule ISX: the drug downregulates oncogenic super enhancers (such as e.g. N-MYC) and converts aggressive Neuroblastoma cells into a more benign form.

## INTRODUCTION

Cellular fates and identities are implemented by chromatin-based core regulatory circuits (CRCs) controlling the expression of lineage-determining transcription factors (TFs). These circuits are often self-stabilizing as they induce transcriptional targets which reinforce the CRC. This re-inforcement is furthermore amplified by the oncogenic TF MYC, which physically associates with CRC genes and potentiates their transcriptional output (1–3). Key to CRC regulation are super enhancers (SEs), chromatin regions with broad and strong histone H3 acetylation at lysine 27 (H3K27ac) (4,5). Not only in normal cells, but also in cancer cells, SEs orchestrate CRCs and therefore determine the transcriptional cancer subtype (6). Such molecular cancer subtypes have been delineated for many malignancies and can possess prognostic as well as predictive impact.

One such malignancy is neuroblastoma (NB), a tumor of the peripheral adrenergic nervous system, which is thought to develop from embryonic neural crest (NC) cells (7). NB typically affects infants and younger children and represents the most frequent tumor diagnosed within the first year of life (8). However, adolescents and young adults can also suffer from this disease and increased age is an established risk factor of this malignancy. Mechanistically, NB is characterized by a blocked terminal differentiation caused by oncogenes, such as e.g. gene-amplified *MYCN* arresting embryonic NC cells during their differentiation along the sympathoadrenal lineage. The mutational burden of NB is comparatively low but certain genes are subject to oncogenic changes such as *ATRX* or the *ALK* kinase (9,10). NB is different from many other cancer types in that its clinical presentation can be extremely heterogeneous: While high risk NB (International Neuroblastoma Staging System (INSS)-stage 4) is an aggressive disease with an overall 5-year survival of only about 30% in *MYCN*-amplified patients, stage 4s NB can spontaneously regress and often does not even require treatment (5-year survival rates of more than 90%) (11). Despite current multimodal treatment approaches, the successful clinical therapy of high risk NB patients is still a challenge, and a better understanding of the heterogeneity of the disease and the development of novel treatment options are mandatory. Two SE-driven transcriptional subtypes have been identified in this disease, which are characterized either by a partial commitment towards the adrenergic lineage (the ADRN subtype) or by an undifferentiated, mesenchymal state (the MES subtype) (12). ADRN and MES states are dynamic and ADRN cells could be converted into MES cells by the expression of defined transcription factors such as *PRRX1* or *NOTCH3* (13,14). Subsequent single cell analyses however suggested that the ADRN subtype might be the prevailing subtype in patients (15,16).

Here, we describe the therapeutic targeting of the SE-amplifying N-MYC TF and the SE landscape in NB cells by the widely used pro-neural substance ISX (17–21). This compound reduced H3K27ac levels at ADRN SEs, resulting in the downregulation of potent NB oncogenes such as *MYCN*, *PHOX2B* and *ALK*. Subsequently, cells were pushed into a MES-like state which shared combined transcriptional features of the MES subtype and ganglioneuroma (GN), a benign tumor closely resembling the neural crest. This small molecule-mediated interconversion of ADRN cells into a MES/GN state was associated with normalizing changes in the global chromatin accessibility landscape, growth arrest and cell death. ADRN subtype cells were particularly vulnerable to this molecular subtype switch, revealing a druggable feature to be exploited in future therapeutic approaches.

## MATERIALS AND METHODS

### Cell lines

IMR32 and SH-SY5Y were purchased from ATCC. GIMEN, NBL-S, LAN-1, SKNBE(2) and NGP cell lines were purchased from DSMZ. GICAN and SHEP lines were kindly provided by Jindrich Cinatl. All cell lines were cultured in Dulbecco's modified Eagle's medium (DMEM (high glucose plus glutamine and pyruvate), Invitrogen) supplemented with 10% fetal bovine serum (FBS) and 1% penicillin/streptomycin at 37°C with 5% CO_2_. If not otherwise stated, serum concentrations were reduced to 0.5% during experiments for all cell types. All cells were regularly checked for mycoplasma contamination.

### Reagents

ISX (= ISX-9) was purchased from Biomol and synthesized in-house by Michael Daude and Wibke Diederich (Core Facility Medical Chemistry and Department of Medicinal Chemistry).

### Colony assays/cell titer assays

Cells were plated and cultured for 4–5 days in the presence of DMSO or ISX (20 μM) in 0.5% FBS-containing growth medium. Subsequently, cells were fixed with 4% formaldehyde/PBS at RT for 10 min, followed by a PBS wash. Then, 10% Giemsa solution was added and cell were stained for 15 min at RT, followed by several washing steps with water to remove excess staining solution. Afterwards, culture plates were air-dried. Cell titer assays were done by seeding 5.000 cells in each well of a white 96well plate with clear bottom, followed by treatment with DMSO or ISX for 4–5 days in 5% FBS-containing growth medium. Subsequently, cell titers were determined using the Cell Titer Glo assay kit (Promega).

### ChIPseq

ChIP was conducted essentially as described (22). Briefly, cells were treated with 20 μM ISX or DMSO for 8 h and incubated with 1% formaldehyde for 10 min at room temperature, washed twice with ice-cold PBS, and harvested with a rubber policeman. Pellets were either shock-frozen in liquid nitrogen for storage or directly resuspended in 1 ml lysis buffer I (5 mM PIPES pH 8.0, 85 mM KCl, 0,5% (v/v) NP40) per 2 × 10^7^ cells and incubated on ice for 20 min. The pellets were then incubated in 1 ml lysis buffer II (10 mM Tris–HCl pH 7.5, 150 mM NaCl, 1% (v/v) NP40, 1% (w/v) sodium deoxycholate, 0.1% (w/v) SDS, 1 mM EDTA) per 2 × 10^7^cells on ice for 10 min. Chromatin was sheared with a Branson S250D ultrasound disintegrator equipped with a microtip. Pre-clearing was performed with 100 μl of IgG-linked blocked protein A sepharose beads (Cytiva) per milliliter of soluble chromatin for 45 min at 4°C with rotation. 1% of each sample was used as the input. 300 μl of soluble chromatin and 2 μg of antibody (see supplemental section for specific antibodies used) were incubated overnight at 4°C with rotation. 50 μl of blocked protein A sepharose beads were added and incubated for 30 min at 4°C with rotation. The beads were once washed with wash buffer I (20 mM Tris pH 8.1, 150 NaCl, 1% (v/v) Triton X-100, 0.1% SDS, 2 mM EDTA), once with wash buffer II (20 mM Tris pH 8.1, 500 mM NaCl, 1% Triton X-100, 0.1 SDS, 2 mM EDTA), and twice with wash buffer III (10 mM Tris pH 8.1, 250 mM LiCl, 1% NP40, 1% sodium deoxycholate, 1 mM EDTA), keeping the samples on ice. Two washes with 10 mM Tris pH8.0 buffer (Qiagen) at room temperature followed. 200 μl of elution buffer (0.1 M NaHCO_3_, 1% SDS) were added to each sample, and the samples were vigorously agitated for 15 min. The supernatants were collected, and elution was repeated, finally combining the supernatants for each sample. Crosslinks were reversed at 65°C overnight, and DNA was purified using a PCR purification kit (Qiagen). Elution was performed twice with 30 μl each of buffer EB from the kit. ChIPSeq libraries were prepared from purified ChIP DNA with the Microplex Library Preparation Kit (Diagenode) according to the manufacturer's instructions. The quality of sequencing libraries was controlled on a Bioanalyzer 2100 using the Agilent High Sensitivity DNA Kit (Agilent). Pooled sequencing libraries were sequenced on a NextSeq 550 platform (Illumina) with 50 bases single reads. Sequencing data were aligned to the human genome GRCh38 (provided by Ensembl (23) version 104). Paired end data was aligned with Subread 2.0.3. For plotting, only R1 reads were used (24). Peak calling was performed using MACS2 (25) version 2.2.7.1. For comparative analyses, ChIPseq data was normalized to the sequencing depth of a different-species spike-in (here: untreated mouse 3T3 cells; 10% (v/v) of 3T3 chromatin was added to each IMR32 chromatin sample prior to ChIP). Specifically, the raw ChIP coverage signal was scaled by multiplication with the number of mouse reads (/1e6). Graphic visualization of ChIPseq tracks was done with IGV (Integrated Genome Viewer) software.

### TF motif search

HOMER 4.11 (26) was employed for known motif identification using findMotifsGenome.pl with the options mask and size 200 on *de novo* ATACseq peaks identified after ISX treatment versus vehicle control (DMSO).

### Ranking of SEs (ROSE)

ROSE (4,27) including the hg38 update was executed with python 2.7.16. Briefly, genomic regions enriched for the histone mark H3K27ac were stitched together if they were within 12.5 kb of each other and stitched regions were ranked by their read density. The threshold where read density began to increase strongly was set as the point where the tangent line with a slope of one is found, and enhancers located above were designated as super enhancers. The input GFF file was created from the H3K27ac peaks in DMSO-treated cells identified by MACS2, and super enhancers were ranked using the aligned reads from the H3K27ac DMSO and ISX ChIPseq samples.

### HDAC assays

The HDAC assay with recombinant enzymes was performed at Reaction Biology Corp. (Malvern, USA). The fluorescent assay used 7-Amino-4-methylcoumarin (AMC) coupled to an acetylated tetrapeptide comprising the p53 residues 379–382 (RHKKAc-AMC) as a substrate. Trichostatin A (TSA) was used as positive control. Assays to determine cellular HDAC activity were done using the HDAC-GLO I/II kit from Promega.

### ATACseq

IMR32 cells were treated with DMSO/ISX (20 μM) for 48 h in 0.5% FBS-containing media. Attached cells were washed and treated with DNaseI (200 U/ml in 250 mM MgCl_2_/50 mM CaCl_2_) for 30 min at 37°C. Subsequently, cells were washed with PBS, trypsinized and 100 000 cells of each condition were sent to Active Motif for ATACseq custom service.

### Xenograft experiment

SH-SY5Y cells were treated with DMSO or ISX (20 μM) for 48 h in culture (0.5% FBS-containing medium). Subsequently, 1 × 10^7^ cells of each condition were resuspended in DMEM and injected s.c. into the flanks of female athymic nude mice. Tumor development was followed by caliper measurements with volumes calculated by the formula ‘length × width^2^)/2’. The length represented the longer axis and the width represented the shorter axis of the tumor. At the experimental endpoint mice were euthanized and tumors were removed. The study was approved by the regional agency on animal experimentation (Regierungspräsidium Giessen).

### Statistics and data accessibility

Statistical comparisons were made of n≥3 experiments using an unpaired two-tailed Student's t-test (MS Excel or GraphPad Prism). Significances were indicated as ns (not significant; *P* > 0.05), *P < 0.05, **P < 0.01, ***P < 0.001. Gene signatures used in this manuscript have been explained summarized in Supplementary Table S1. Kaplan–Meier curves, gene expression analyses, PCAs, gene correlations from public datasets were done using the R2: Genomics Analysis and Visualization Platform (http://r2.amc.nl). Data can be accessed through Array Express: RNAseq: E-MTAB-10249 (IMR32; previously published in: (28)); E-MTAB-11915 (SH-SY5Y). ChIPseq: E-MTAB-11652 (IMR32); ATACseq: E-MTAB-11633 (IMR32). The graphical abstract was created with BioRender.com.

## RESULTS

### Key NB oncogenes belong to the ADRN subtype

**Figure 1.**
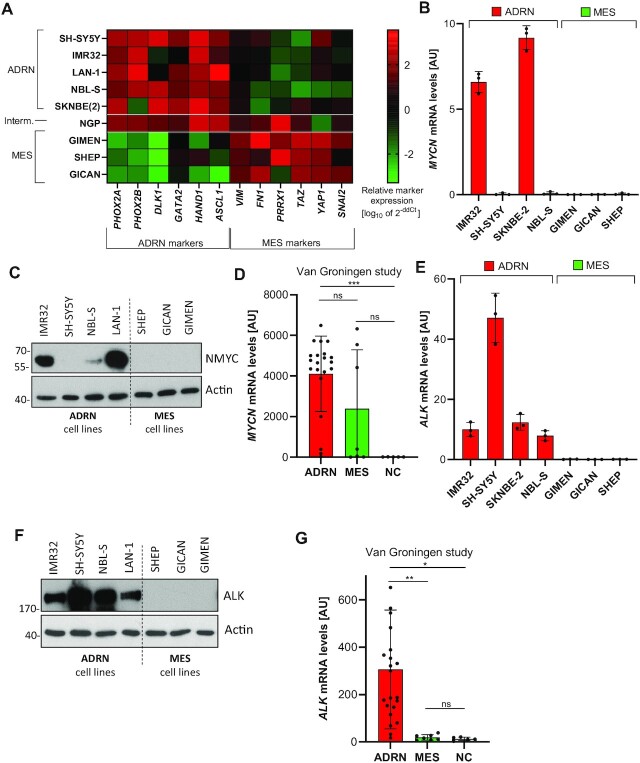
N-MYC and ALK are NB subtype-specific oncogenes. (**A**) Heatmap depicting expression (qPCR) of ADRN/MES marker genes in a panel of different NB cell lines. Interm. = intermediate. Colors represent the mean expression of *n* = 3 experiments. (**B**) *MYCN* mRNA expression in ADRN and MES cell lines as determined by qPCR. Mean of *n* = 3 (±SD) experiments. (**C**) N-MYC protein levels in ADRN and MES cell lines. Shown is one representative result of *n* = 3 experiments. (**D**) *MYCN* mRNA expression in a cellular model of MES/ADRN/NC differentiation (14). MES (mesenchymal; *n* = 7); ADRN (adrenergic; *n* = 22); NC (neural crest; *n* = 5). Shown is the mean ±SEM. (**E**) *ALK* mRNA expression in ADRN and MES cell lines (qPCR). Mean of *n* = 3 ± SD. (**F**) ALK protein levels in ADRN and MES cell lines. Shown is one representative result of *n* = 3. (**G**) *ALK* mRNA expression in a cellular model of MES/ADRN/NC differentiation (14). MES (mesenchymal; *n* = 7); ADRN (adrenergic; *n* = 22); NC (neural crest; *n* = 5). Shown is the mean ± SEM.

As previously reported by others (12,14), we could separate various NB cell lines into ADRN and MES groups based on their subtype-specific marker expression (Figure 1A). As recent literature had already indirectly implied (29), we found that the expression of the central NB oncogene *MYCN* was restricted to the ADRN class of cell lines as verified on mRNA (Figure 1B) as well as protein level (Figure 1C). It should hereby be noted that NBL-S cells expressed elevated N-MYC protein levels despite low *MYCN* mRNA. In addition, SH-SY5Y are known to express increased c-MYC (MYC) protein, ostensibly compensating for the lack of *MYCN* amplification. Thus, the majority of ADRN cell lines displayed upregulated MYC levels, which was not observed in MES lines. Moreover, a recent study of 34 NB cell lines and primary cell samples (14) also revealed the highest *MYCN* expression in the ADRN group, with a trending decrease towards MES and a significant drop towards neural crest (NC) (Figure 1D). Besides *MYCN*, the *ALK* kinase represents another cardinal oncogene in NB (9). In line with *MYCN*, also high *ALK* expression was selectively encountered in the ADRN subset of cell lines, both on mRNA (Figure 1E) and on protein levels (Figure 1F). Again, a publicly available dataset on ADRN/MES/NC samples (14) revealed significantly elevated *ALK* expression selectively in the ADRN versus MES and NC samples (Figure 1G). Supporting this finding, an experimentally induced ADRN-to-MES transition in SK-N-BE(2)C cells (14) was accompanied by the concomitant downregulation of *ALK* (as well as *PHOX2A*, another ADRN marker) expression while cells adopted the MES phenotype (Supplementary Figure S1A, B). Albeit *MYCN* was unaffected in the latter assay (Supplementary Figure S1C), our data supported the overall view of *MYCN* and *ALK* as ADRN-enriched oncogenes in NB.

### ISX abrogates ALK and N-MYC functions in NB cells

**Figure 2.**
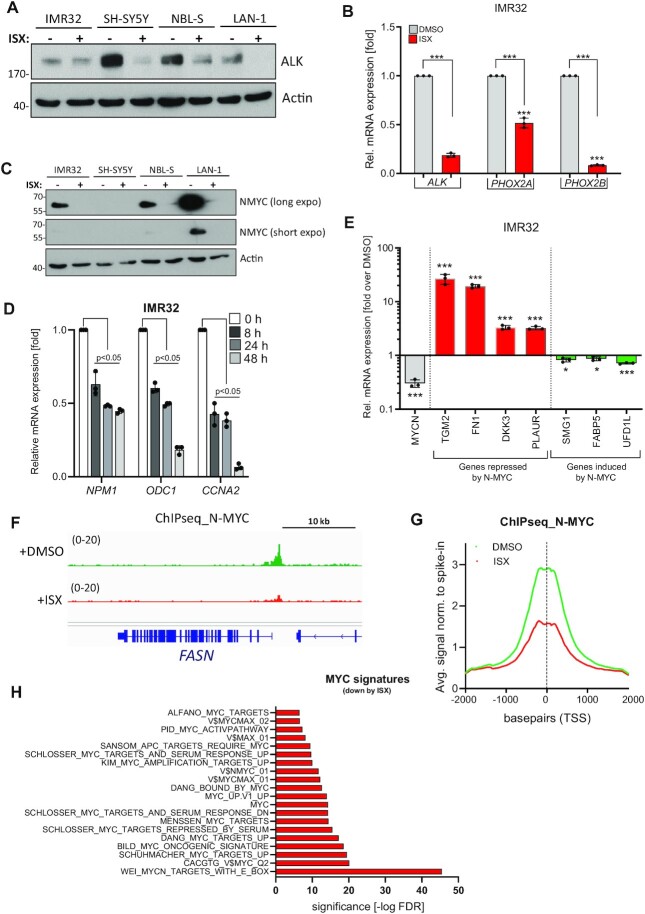
Key NB oncogenes are downregulated by ISX. (**A**) Change of ALK protein levels in ADRN cell lines upon treatment with ISX (20 μM) for 48 h. Shown is one representative result of *n* = 3. (**B**) Relative mRNA expression (*ALK, PHOX2A, PHOX2B*) in IMR32 cells exposed to DMSO or ISX (20 μM, 48 h) as determined by qPCR. Mean of *n* = 3 ± SD. (**C**) Change of N-MYC protein levels in human NB cell lines upon treatment with ISX (20 μM) for 48 h. Shown is one representative result of *n* = 3. (**D**) Relative mRNA expression of canonical MYC/N-MYC target genes in IMR32 cells treated with 20 μM ISX for the indicated times as determined by qPCR. Shown is the triplicate measurement of one experiment of *n* = 2. (**E**) Relative mRNA expression (qPCR) of N-MYC target genes in NB. IMR32 cells were treated with 20 μM ISX for 48 h. Mean of *n* = 3 (±SD) experiments. (**F**) Spike-in-normalized ChIPseq track of N-MYC binding at the *FASN* promoter (MYC target gene) in IMR32 cells exposed to DMSO or ISX (20 μM) for 8 h. (**G**) Average plots of N-MYC (spike-in-normalized) chromatin binding (ChIPseq reads) in IMR32 cells treated with DMSO or ISX (20 μM) for 8 h. Reads are centered on TSS (bp 0). (**H**) MYC-related transcriptional signatures (RNAseq, downregulated genes, cut-off 1.5-fold) from IMR32 cells treated with ISX (20 μM) for 48 h. DMSO-treated cells served as control.

We serendipitously observed that treatment of cells with the widely used pro-neural small molecule ISX (17) potently abrogated ALK expression, verified both on protein (Figure 2A, Supplementary Figure S2A, B) and mRNA level (Figure 2B). Moreover, ISX also significantly suppressed the gene expression of the ADRN-restricted oncogenes *PHOX2A* and *PHOX2B* (30) (Figure 2B). In addition, N-MYC protein levels were also strongly reduced upon ISX exposure in several cell lines (Figure 2C) and in a concentration-dependent mechanism (Supplementary Figure S2C). As a result, canonical N-MYC/MYC target genes such as *NPM1*, *ODC1* or *CCNA2* were found to be significantly downregulated by ISX in a time-dependent manner (Figure 2D), which was kinetically comparable with declining N-MYC protein levels over time (Supplementary Figure S2D). Furthermore, NB-specific negative N-MYC target genes (e.g. *TGM2, DKK3*) were de-repressed by ISX whereas positive N-MYC target genes (e.g. *FABP5, UFD1L*) were concomitantly suppressed (Figure 2E), overall indicative of N-MYC inhibition by ISX. In order to shed more light on this issue, we performed spike-in-normalized chromatin immunoprecipitation followed by DNA sequencing (ChIPseq) using an N-MYC-specific antibody in IMR32 cells. These experiments revealed that endogenous N-MYC binding was grossly reduced at the promoter of known target genes such as e.g. *FASN* or *NCL* upon ISX treatment already after 8h of treatment (Figure 2F and Supplementary Figure S2E). Reduced N-MYC binding at target genes could also be verified by ChIP-qPCR (Supplementary Figure S2F). These locus-specific findings could be extended in a genome-wide analysis demonstrating an ISX-dependent global reduction of N-MYC association with transcription start sites (TSSs) (Figure 2G). Consequently, RNAseq analyses to investigate transcriptome-wide changes significantly retrieved many MYC-related signatures in the ISX-downregulated genes (Figure 2H). Additionally, ISX-downregulated gene sets included several cell cycle-related signatures (Supplementary Figure S2G) whereas upregulated gene sets were dominated by chromatin-associated signatures (histone modifications, chromatin remodeling) (Supplementary Figure S2H). In summary, our data provided evidence for ISX being a potent inhibitor of key ADRN-restricted NB oncogenes such as *ALK*, *PHOX2B* and *MYCN* and implied chromatin-related processes in this inhibition.

### ISX induces NB subtype switching

**Figure 3.**
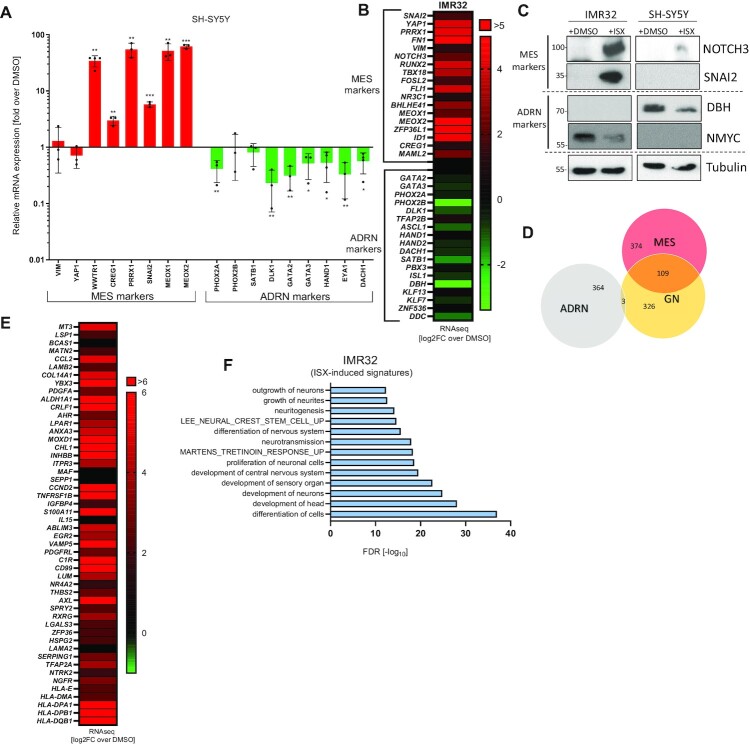
ISX induces NB subtype switching. (**A**) Relative mRNA expression of ADRN/MES marker genes in SH-SY5Y cells treated with DMSO or ISX (20 μM) for 48 h (qPCR). Shown is the mean of *n* ≥ 3 ± SD. (**B**) Heatmap depicting ISX (20 μM)-induced changes (log_2_-fold over DMSO) of ADRN/MES marker genes in IMR32 cells (RNAseq). (**C**) Change of selected ADRN (DBH, N-MYC) and MES (NOTCH3, SNAI2) marker proteins in two NB cell lines upon treatment with ISX (20 μM) for 48 h. Shown is one representative result of *n* = 2. (**D**) Venn diagram showing overlaps between the ADRN/MES/GN gene signatures. Numbers indicate number of transcripts. (**E**) Heatmap depicting ISX-induced changes (log_2_-fold over DMSO) of GN marker genes in IMR32 cells (RNAseq). Shown are 50 GN genes not present in the MES signature. (**F**) ISX (20 μM, 48 h)-induced transcriptomic terms in IMR32 cells (RNAseq).

In light of the effects of ISX on ADRN oncogenes we wanted to investigate the impact on ADRN/MES phenotypes in more detail. To this end, we treated NB cells with ISX and measured a panel of well-established MES/ADRN marker genes (12,14). As can be seen in Figure 3A, ISX strongly upregulated MES-associated transcripts such as *WWTR1 (TAZ), CREG1, PRRX1, SNAI2* and *MEOX2* in SH-SY5Y cells while downregulating ADRN markers like *PHOX2A, DLK1, GATA2* or *HAND1*. Moreover, global transcriptomics in a second cell line (IMR32) cells also confirmed a striking upregulation of MES markers with a concomitant downregulation of ADRN genes upon ISX treatment (Figure 3B). A comparable pattern was verified in SKNBE(2) cells, another ADRN cell line (Supplementary Figure S3A), but was interestingly absent in the MES cell line GIMEN (Supplementary Figure S3B). The ISX-mediated switch of NB subtypes could not only be observed on the transcript level, but also be verified on the protein level for the ADRN markers DBH and N-MYC and the MES markers NOTCH3 and SNAI2 (Figure 3C). In total, ISX induced almost half of the genes contained in the MES signature in IMR32 cells (*P = 0.035* (hypergeometric test); Supplementary Figure S3C) with <10% of ADRN genes being induced (Supplementary Figure S3D), demonstrating a clear MES-directed bias among the upregulated genes. In contrast, a significant contribution to the ADRN gene set was observed in the ISX-downregulated genes (*P* = 4.6 × 10^−^^3^ (hypergeometric test); Supplementary Figure S3D).

The loss of ADRN markers and the gain of MES markers could be observed already after 4–8 h of ISX treatment (Supplementary Figure S3E, F). Transcriptome-wide analyses in forms of scatter plot and PCA also verified an upregulation of MES features with a concomitant drop in expression of ADRN markers (Supplementary Figure S3G) and a global transcriptional shift of the treated cells from an ADRN towards a MES/NC state (Supplementary Figure S3H). In further support of these findings of an ADRN-to-MES switch, the top 500 ISX-upregulated genes (IMR32) were also found to be time-dependently stimulated in a published data set of experimental MES subtype induction (14) (Supplementary Figure S3I). In addition to the reported MES markers, ISX transcriptionally upregulated also pro-differentiating neurotrophin genes (*NGF, NT3*) as well as the p75 NGF receptor (*NGFR*) in IMR32 cells (Supplementary Figure S4A–C). Noteworthy, *NGF*, *NT3* and *NGFR* transcripts were found to be elevated in MES/NC versus ADRN samples (Supplementary Figure S4D–F), as was that of the Hedgehog target gene *GLI1* (Supplementary Figure S4G), which we previously reported to be induced by ISX (28).

In further investigations we observed that a gene signature derived from the benign tumor ganglioneuroma (GN, for list see Supplementary Table S1) (31) possessed a considerable transcriptional overlap with the MES signature, but not with the ADRN subset of genes (Figure 3D). In fact, ISX induced roughly half of the benign GN signature in IMR32 cells (Supplementary Figure S4H), a finding which might explain why the MES signature was associated with a significantly better prognosis in NB patients (Supplementary Figure S4I). In addition, when randomly selecting 50 GN genes not being part of the MES gene set, we found that ISX strongly induced most of them (Figure 3E). In total, ISX upregulated half of both, the MES as well as the GN gene set, suggesting that ISX induced a mixed MES/GN (‘MES-like’) state. In agreement with GN being a well-differentiated benign tumor, ISX highly significantly upregulated transcriptional terms such as *‘differentiation of cells’*, *‘development of head’* or *‘development of neurons’* in RNAseq experiments using IMR32 cells (Figure 3F). In conclusion, ISX provoked a switch from ADRN to a mixed MES/GN subtype which was associated with the induction of differentiation genes.

### ADRN cells are particularly sensitive against ISX-mediated subtype reprogramming

**Figure 4.**
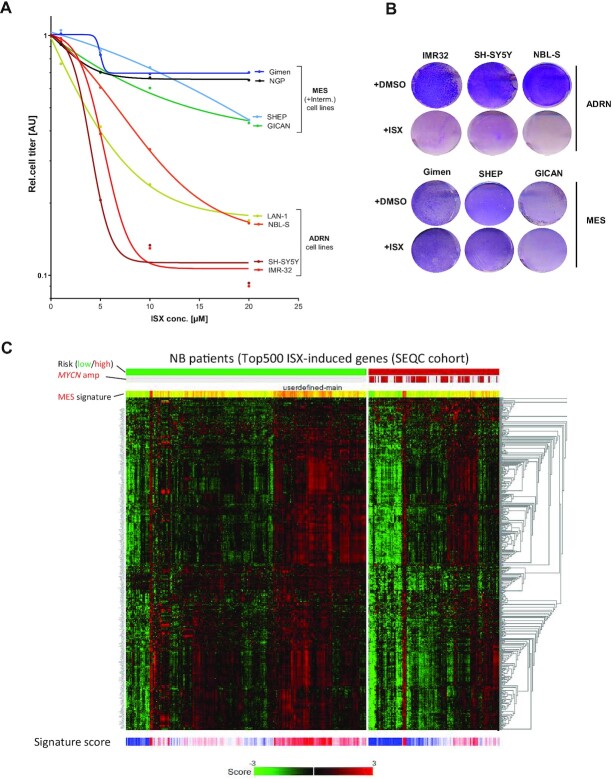
ISX effects are selective for the ADRN subtype. (**A**) Cell titer assays of ADRN/MES NB cell lines treated with the indicated concentration of ISX for 4 days. Each data point is the mean of *n*≥ 3 experiments. Non-linear line fit is log fit/variable slope (four parameters). (**B**) Colony stain (Giemsa, blue) of NB cells treated with ISX (20 μM) for 4–5 days. Shown is one representative of *n* = 2. (**C**) Heatmap depicting the top 500 ISX-induced genes (derived from IMR32 cells exposed to 20 μM ISX for 48h) in low- and high-risk NB patients.

Since ISX was capable of inducing differentiation terms in NB cells, we assumed that this compound would also result in overall growth retardation. Therefore, we next queried the proliferative response of cells from the ADRN versus MES subtypes (Figure 1A) against ISX exposure. Interestingly, we observed that ADRN cell lines were far more susceptible to the growth-inhibitory properties of ISX when compared to MES cell lines, which were almost non-sensitive (Figure 4A, B). These results showed that ADRN cells were particularly vulnerable for the effects of ISX. Since ADRN cells represent the vast majority of NB subtypes in patients (15,16), this finding might open new possibilities of therapeutic targeting. In fact, the ISX-induced gene signature (top 500 genes) was clearly over-represented in low-risk versus high-risk NB patients (Figure 4C), which was in line with our findings that ISX suppressed key NB oncogenes. Interestingly, expression of ISX-induced genes was prominently found in about 40% of low risk patients (Figure 4C). We wondered whether these patients represent an own group and we therefore performed *k*-means clustering on a publicly available NB transcriptome data set (SEQC cohort, *n* = 498 patients (32)). This analysis revealed three distinct transcriptional groups (group 1–3 (not to be confused with INSS risk stages); Supplementary Figure S5A) and patient transcriptomes of these groups could readily be segregated in principle component analysis (PCA; Supplementary Figure S5B). Intriguingly, group 3 patients were enriched for stage 4 (high-risk) disease whereas groups 1 and 2 contained many low-risk stages (stages 1, 2, 4s) (Supplementary Figure S5C), a finding also reflected in a worse overall survival of group 3 patients in comparison to groups 1/2 (Supplementary Figure S5E). Interestingly, ISX-induced genes were predominantly associated with group 2 patients (Supplementary Figure S5E), suggesting that the aforementioned patient group (Figure 4C) indeed represented group 2 patients. Similar findings were also made with an independent set of patients (Kocak cohort; n = 649; Supplementary Figure S5F) (33).

### ISX affects histone acetylation and promotes super enhancer weakening

**Figure 5.**
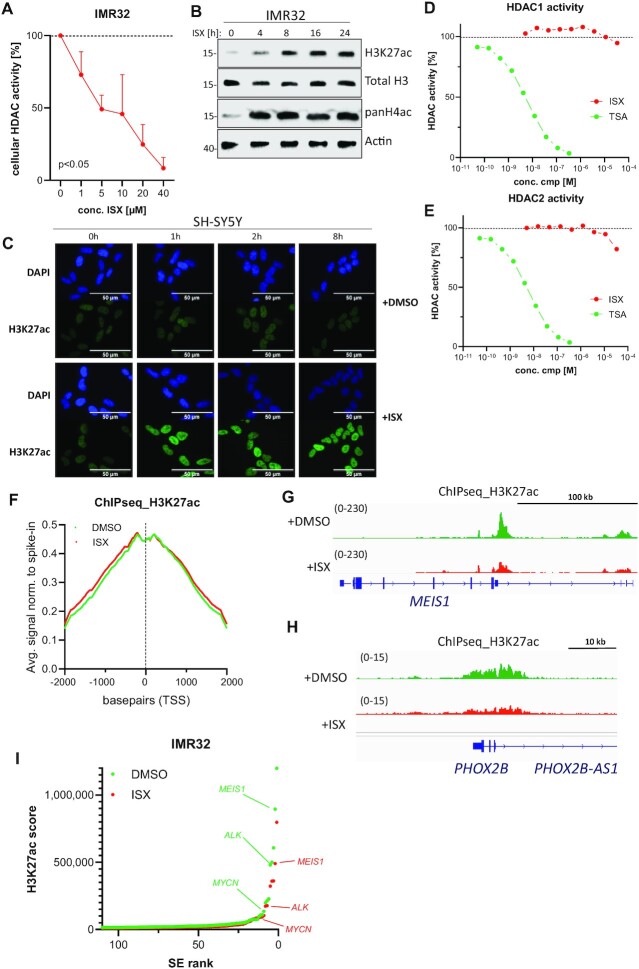
ISX exerts disparate effects on histone acetylation. (**A**) Cellular HDAC (classI/II) activity in IMR32 cells exposed to increasing concentrations of ISX for 1h. Shown is one representative experiment of n = 2 measured in triplicate (mean ± SD). (**B**) Western blot of ISX-treated IMR32 cells. ISX (20 μM) was applied for the indicated time periods. (**C**) H3K27ac immunofluorescence (green) of DMSO- or ISX (20 μM)-treated SH-SY5Y cells. Nuclei appear in blue (DAPI). Scale bar 50 μm. (**D**) HDAC enzyme assay using recombinant human HDAC1. Data point are means of *n* = 2 measurements. Trichostatin A (TSA) was used as positive control. (**E**) HDAC enzyme assay using recombinant human HDAC2. Data point are means of *n* = 2 measurements. Trichostatin A (TSA) was used as positive control. (**F**) Spike-in-normalized average plots of H3K27ac ChIPseq reads from IMR32 cells treated with DMSO or ISX (20 μM) for 8 h. Reads are centered on TSS (bp 0). (**G**) Spike-in-normalized H3K27ac ChIPseq track of the *MEIS1*-associated super enhancer. IMR32 cells were treated with DMSO or ISX (20 μM) for 8 h. (**H**) Spike-in-normalized H3K27ac ChIPseq track of the *PHOX2B*-associated super enhancer. IMR32 cells were treated with DMSO or ISX (20 μM) for 8 h. (**I**) Super enhancer (SE) ranking (ROSE) according to spike-in-normalized H3K27ac signal (IMR32 treated with DMSO or ISX (20 μM) for 8 h).

Next, we were interested to shed more light on the underlying molecular mechanism of ISX. We recently reported on ISX as a Hedgehog/GLI1-inducing substance and its impact on histone acetylation (28). In addition, MYC (or N-MYC, which is regulated by ISX) had previously been shown to be a major positive regulator of histone acetylation (34). Hence, we first studied HDAC activity and histone acetylation in ISX-treated NB cells. In line with our previous results from various non-neuronal cell types, we found that ISX strongly reduced cellular HDAC activity also in cultured NB cells (Figure 5A and Supplementary Figure S6A). Furthermore, it strongly upregulated histone H3 and H4 acetylation in both Western blot (Figure 5B) and immunofluorescence assays (Figure 5C), underscoring the previous view of ISX as a potential (class I) HDAC inhibitor. Surprisingly however, ISX failed to block HDAC1 or HDAC2 activity in assays utilizing recombinant enzyme, in contrast to the well-established pan-HDAC inhibitor trichostatin A (TSA) which fully abrogated HDAC enzymatic activity several orders of magnitude below the highest concentration of ISX (Figure 5D, E). These experiments pointed to ISX being an inducer of global histone acetylation without being a direct HDAC inhibitor.

In order to investigate the impact of ISX on histone acetylation in more detail, we performed spike-in-normalized ChIPseq studies using antibodies against H3K27ac in the human NB cell line IMR32. We specifically chose a shorter treatment time window (8 h) in order to avoid many secondary changes. At this time point, N-MYC target genes were starting to decline (Figure 2D) but considerable N-MYC protein levels were still present (Supplementary Figure S2D). In agreement with our previous findings (Figure 5B, C), ChIP investigations confirmed a slight but widespread increase of global histone acetylation by ISX, which mostly occurred outside of TSS regions (Figure 5F).

Since the ADRN/MES subtypes are defined by the activities of their respective SEs and the latter elements are labelled by strong H3K27 acetylation, we investigated this mark at key CRC member genes. Despite a global increase in histone acetylation by ISX (Figure 5B, C, F), compound treatment clearly reduced H3K27 acetylation at the *MEIS1* and *PHOX2B* (Figure 5G, H) SE regions. This observation could also be verified by ChIP-qPCR (Supplementary Figure S6B). Moreover, plotting all SE regions (ROSE algorithm; (27)) in IMR32 cells according to their H3K27ac signal revealed a broad downregulation of SE intensities by ISX (Figure 5I). In addition, the drug-induced alterations in H3K27 acetylation were paralleled by a loss of N-MYC binding at super enhancers (Supplementary Figure S6C–E), strongly implying that the drug-induced loss of SE-bound N-MYC might be causal for the locus-specific decrease in H3K27 acetylation. The latter effect also applied to normal enhancers (Supplementary Figure S6F).

In summary, we observed a slight but widespread induction of histone acetylation by ISX, which was most likely caused by its HDAC-inhibitory capability. Moreover, this compound also led to a genome-wide loss of N-MYC DNA binding, the latter being also evident at SEs. Most importantly, these findings give a direct mechanistic explanation for the loss of SE-driven gene expression and the subtype switch described before.

### ISX evokes chromatin alterations associated with differentiation and low-risk NB

**Figure 6.**
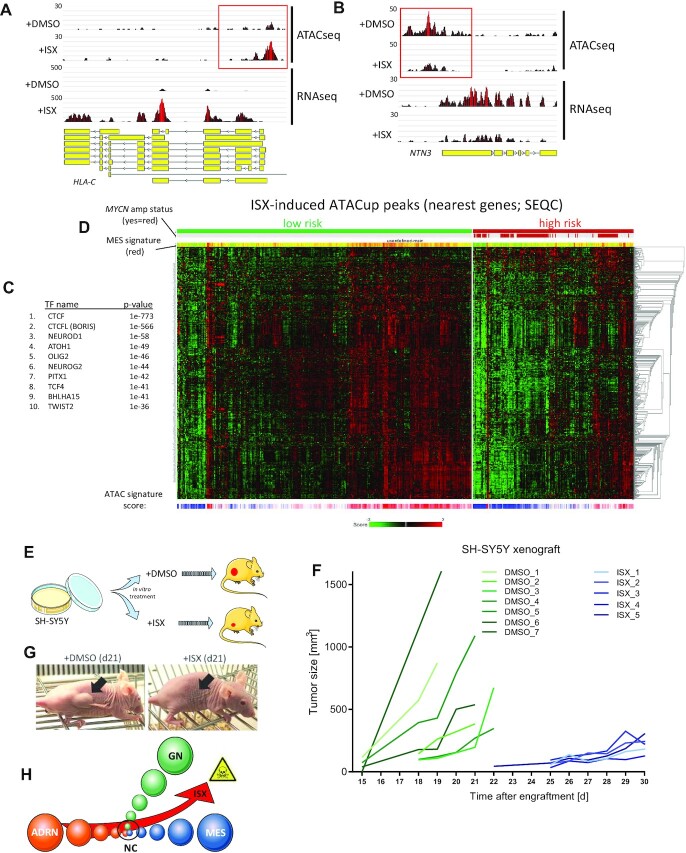
ISX installs a long-lasting chromatin accessibility landscape found in low risk NB. (**A**) Example of an ATACseq peak upregulated by ISX (20 μM, 48 h). Shown is the *HLA-C* gene locus in IMR32 cells. (**B**) Example of an ATACseq peak downregulated by ISX (20 μM, 48 h). Shown is the *NETRIN3 (NTN3)* gene locus in IMR32 cells. (**C**) Transcription factor (TF) binding motif analysis (Homer) of ISX-induced ATAC peaks in IMR32 cells (nearest genes of ATACup peaks). (**D**) Heatmap depicting nearest genes to ISX-induced ATAC peaks (ATACup) in the SEQC cohort of NB patients (clustered into low/high risk patients). (**E**) Schematic outline of the SH-SY5Y xenograft experiment. Cells were pre-treated with compounds (DMSO or 20 μM ISX) in cell culture for 48 h, followed by injection of similar cell numbers into the flanks of nude mice. (**F**) SH-SY5Y xenograft tumor growth over time. Day 0 represents the day of injection. Each line represents one animal. Start of line denotes first measurable time point. Only those animals are depicted which displayed measurable tumor growth. (**G**) Two representative animals at day 21 after inoculation. The black arrows depict the injection sites. (**H**) Schematic drawing depicting the overall findings of this manuscript. NB cell lines, originally arising from the neural crest (NC), belong to two dynamic subtypes (ADRN and MES). In addition, the benign Ganglioneuroma (GN) represents a differentiated form of a NC-derived sympathetic tumor. ISX can induce the transition from the ADRN subtype to a mixed MES/GN (MES-like) subtype, which is associated with growth retardation and NB cell death.

Cell fate decisions are controlled by SEs but reflected and stably installed by changes in global chromatin accessibility (35,36). Hence, in order to investigate the genome-wide chromatin alterations associated with the ISX-induced ADRN-to-MES-like switch in more detail we performed ATACseq (Assay for Transposase-Accessible Chromatin using sequencing) studies in the ADRN cell line IMR32. These experiments revealed that ISX induced about 1.5-fold more ATAC peaks (ATACup peaks) than it repressed (ATACdown peaks) (Supplementary Figure S7A). Roughly 1600 genes were associated with these ISX-altered ATAC peaks, of which about 300 were also transcriptionally regulated by the drug. Upregulated ATAC peaks included for instance the *HLA-C* gene locus (Figure 6A) whereas the group of genes associated with ISX-downregulated ATAC peaks encompassed important NB disease genes such as *MYCN, ALK* or *NTN3* (37), which were accordingly transcriptionally repressed by the drug (Figure 6B and Supplementary Figure S7B).

Surprisingly however, in its entirety, the ATACup changes were more often correlated with transcriptional changes (Supplementary Figure S7A), which was in line with the increased histone acetylation levels evoked by the drug (Supplementary Figure S7B). Validating widespread chromatin alterations by ISX, principle component analysis (PCA) could readily separate DMSO- from ISX-treated samples based on the ATACseq results (Supplementary Figure S7D).

A search for TF binding sequences of the ATACup-associated genes revealed high-ranking chromatin and enhancer regulators (CTCF, BORIS) as well as many TFs involved in neuronal differentiation (NEUROD1, ATOH1, OLIG2, NEUROG2) among the top identified motifs (Figure 6C). Accordingly, the ATACup-associated gene sets were significantly enriched for NB-relevant transcriptome terms such as *‘development of neurons’*, *‘neuritogenesis’* or *‘morphogenesis of neurites’* (10) (Supplementary Figure S7E). Furthermore and of translational significance was the fact that the genes located closest to ATACup peaks clearly correlated with the transcriptomes of low-risk (versus high-risk) NB patients (Figure 6D), indicative of a drug-mediated restructuring of the chromatin landscape from a high-to-low risk scenario.

Since ISX exerted widespread chromatin remodeling activity we assumed that these changes might be longer-lasting once they were established in treated cells. In order to investigate whether this was true and whether these effects would be observable also *in vivo*, we decided to pretreat human SH-SY5Y cells for 48 h in culture and then xenotransplant identical numbers of DMSO- or ISX-pretreated cells subcutaneously into nude mice without any further drug treatment (Figure 6E). Indeed, the ISX-pretreated NB cells were significantly growth retarded (Figure 6F) and virtually lacked measurable tumors at time points at which control animals (containing DMSO-pretreated cells) had to be sacrificed due to tumor load (Figure 6G). Despite ISX-pretreated cells resuming their growth after some weeks, it was surprising to note that a mere 48 h-pretreatment period was sufficient to induce an ∼1.5–2 week delay in *in vivo* tumor growth (Figure 6F). Pre-treated tumors eventually reverted back to an ADRN phenotype as evidenced by comparable subtype marker gene expression (Supplementary Figure S7F), histology (Supplementary Figure S7G) and tumor cell proliferation (Ki67, Supplementary Figure S7G, H). Furthermore, the ADRN marker synaptophysin (Supplementary Figure S7I), which was efficiently suppressed by ISX in culture (Supplementary Figure S7J, K) was detected in tumor tissue of both treatment groups at the end of the experiment (Supplementary Figure S7G). In conclusion, short ISX pre-treatment was sufficient to rewire the chromatin landscape and to induce long-lasting anti-proliferative changes in human NB cells resulting in significant retardation of *in vivo* tumor growth.

## DISCUSSION

Cancer, and in particular neuroblastoma (NB), is intimately associated with aberrant cellular differentiation. However, despite the knowledge about super enhancers (SEs) as genomic elements determining cell fate and differentiation, pharmacological approaches targeting SE-controlled core regulatory circuits (CRCs) have been limited but are slowly emerging. In principle, targeting CRCs is an attractive option as the disruption of a single signaling loop would functionally inactivate several key oncogenes in one step. Here, we could demonstrate that the small molecule ISX, best established for its pro-neural differentiation activity (17-21), is a CRC disruptor in adrenergic NB. The underlying mechanism is most likely its N-MYC-inactivating functionality as MYC proteins are known amplifiers of oncogenic CRCs (1-3). The ability to modulate cell lineage-selective SE circuits probably also explains why ISX previously displayed cell type-specific effects in many non-neuronal systems (38–41) and also proved non-toxic *in vivo* in non-cancerous settings (19,21,42). In our hands, ISX reprogrammed NB cells from an ADRN into a MES-like (MES/GN) state which is incompatible with oncogenic growth (Figure 6H). ADRN and MES represent two major molecular subtypes in NB which can transition into each other by the activity of subtype-specific SEs and the resulting set of transcription factors (12-14). Recently, the dual ADRN/MES categorization was further refined into four subtypes including a mesenchymal group with some similarity to Schwann cell precursor cells (43). In light of Schwann cells being one possible cellular origin of GN, the ISX-induced MES/GN signature might as well overlap with some of these newly identified subtypes.

It was intriguing to note that only ADRN cells were sensitive for the growth-inhibitory functions of ISX. We speculate that the downregulation of ADRN-restricted SE activities regulating key ADRN oncogenes such as *MYCN*, *ALK* or *PHOX2B* might account for this subtype specificity. In fact, it was previously shown that ADRN oncogenes affect NB in a subtype-specific manner (43). Several reports associate disease relapse with the MES subtype of NB (43,44) and it will have to be investigated to what extent an ISX-induced signature would contribute to these processes. However, a drug-induced MES signature could also provide clinical benefits as it might sensitize cancer cells for immunotherapy (45). Hence, future investigations on the combination of ISX and immune modulators appear promising.

From a translational or pharmacological point of view, only few compounds have been described to be able to target the oncogenic SE landscape, such as inhibitors of the histone acetyl transferase p300, the histone acetylation reader protein BRD4, histone deacetylase inhibitors, or the cyclin-dependent kinase CDK7 (27,46–49), supporting the view of SEs as structures sensitive to changes in histone acetylation (50,51). Noteworthy however, the majority of the reported drugs appear to possess a different mode of action compared to ISX as they grossly suppress overall transcriptional processes which becomes most pronounced at SE-controlled CRCs. In contrast, ISX elevates global histone acetylation and induces more genes than it represses, thus acting as a transcriptional enhancer in many instances except for the ADRN CRC genes. As such, only ISX but not a CDK7 inhibitor was capable of doing both, suppressing ADRN genes while also inducing MES genes (Supplementary Figure S8A and Figure 3A, B). Hence, although the exact mode of ISX action requires further investigations, it appears fundamentally distinct to that of other SE-targeting compounds.

Overall, it was interesting to see that ISX evoked far reaching changes in the chromatin accessibility landscape of ADRN cells. As chromatin accessibility is considered a defining feature of cell fate (35,36), this observation is perfectly in line with the drug-mediated modulation of CRCs and the resulting subtype switch. Specifically, ISX-treated cells adopted a *de novo* accessibility landscape associated with low-risk NB, a feature which might be supported by the inherent property of ISX to increase histone acetylation by indirectly blocking class I HDACs (this study and (28)). In fact, pharmacological HDAC inhibition has previously been reported to drive NB differentiation (52). The widespread epigenomic alterations induced by ISX probably also explain the long-lasting effects of this drug which allowed us to investigate the *in vivo* behavior of pretreated cells over an extended period of time. These observations could be of high clinical relevance in the future as short treatment periods could potentially result in long-lasting therapeutic effects.

In summary, we provide proof-of-principle that pharmacological CRC reprogramming and NB subtype switching is possible and that it might be harnessed therapeutically in the future in patients suffering from ADRN-dominated NB. Similar concepts might also be applicable to other tumor entities as well, opening new avenues in cancer research and treatment.

## DATA AVAILABILITY

Kaplan-Meier curves, gene expression analyses, PCAs, gene correlations from public datasets were done using the R2: Genomics Analysis and Visualization Platform (http://r2.amc.nl). Data can be accessed through Array Express: RNAseq: E-MTAB-10249 (IMR32; previously published in: (28)); E-MTAB-11915 (SH-SY5Y). ChIPseq: E-MTAB-12344 (IMR32); ATACseq: E-MTAB-11633 (IMR32).

## Supplementary Material

zcad007_Supplemental_Files
