# Supplementary material for: Tumor-suppressive disruption of cancer subtype-associated super enhancer circuits by small molecule treatment
Source: NAR Cancer. 2023 Feb 6;5(1):zcad007. doi: 10.1093/narcan/zcad007 (PMC9900422; doi:10.1093/narcan/zcad007)
Supplement: zcad007_Supplemental_Files [file zcad007_supplemental_files.zip › Koeniger_et_al_Table_S1.pdf]

Gene signatures used in Koeniger et al.

References: ADRN: From Table S2 in Van Groningen et al., 2017
MES: From Table S2 in Van Groningen et al., 2017
GN: Top 500 genes up in GN vs. NB; from Table S1 in Gershon et al.

ADRN	MES	GN
ABCA3	A2M	APOD
ABCB1	ABRACL	PLP1
ABLIM1	ACADVL	CDH19
ACOT7	ACAP2	GPM6B
ACTL6B	ACTA2	ABCA8
ACVR1B	ACTN1	GAS7
ADAM22	ADAM19	FXYD1
ADCYAP1R1	ADAM9	SEMA3B
ADGRB3	ADAMTS5	MBP
ADRBK2	ADGRE5	ERBB3
AGTPBP1	ADGRG6	CRYAB
AHSA1	AEBP1	QKI
AKAP1	AJUBA	AHR
AKAP12	ALDH1A3	CRYAB
ALK	AMMECR1	ALDH1A1
ANK2	ANTXR1	CHL1
ANKRD46	ANXA1	AHNAK
ANP32A	ANXA2	NR4A2
AP1S2	ANXA5	WWTR1
ARHGEF7	ANXA6	GSN
ARL6IP1	APOE	ITGA6
ASCL1	APP	SOX10
ASRGL1	ARHGAP1	DMD
ATCAY	ARHGEF40	LGALS3
ATL1	ARL1	hCG_1998957
ATP6V0E2	ARL4A	F3
ATP6V1B2	ARMCX2	ASPA
AUTS2	ARPC1B	ANXA1
BEND4	ASPH	LAMA2
BEX1	ATP10D	CEBPD
BEX2	ATP1B1	SPTBN1
BIRC5	ATP2B1	S100A10
BMP7	ATP2B4	GATM
BMPR1B	ATP6V0E1	TGFBR2
C11orf95	ATP8B2	CTNNAL1
C14orf132	ATXN1	SLC16A4
C3orf14	B2M	TGFBR2
C4orf48	BAG3	GATM
C7orf55	BGN	C1S
CACNA1B	BMP5	MATN2

CACNA2D2	BNC2	EPB41L2
CADM1	BOC	C10orf56
CAMSAP1	BTN3A2	COBL
CCDC167	C1orf198	ST6GALNAC2
CCND1	C1orf54	CYP1B1
CCNI	C4orf32	RELN
CCP110	C6orf120	STARD13
CCSAP	CALD1	SPARCL1
CD200	CALU	MIA
CDC42EP3	CAPN2	EGFL8
CDCA5	CAPN6	KCTD12
CDKN2C	CBFB	LGALS1
CDKN3	CBLB	ENDOD1
CELF2	CCDC80	C1R
CENPU	CD164	HLA-DPB1
CENPV	CD44	ITGB4
CEP44	CD59	CLIC4
CERK	CD63	CYP1B1
CETN3	CDH11	PRNP
CHGA	CETN2	PROS1
CHGB	CFH	CYR61
CHML	CFI	NR2F2
CHRNA3	CILP	LUM
CKB	CKAP4	MAF
CLASP2	CLIC4	GPR126
CLGN	CMTM3	HLA-DPB1
CRH	CMTM6	EGR3
CRMP1	CNN3	IGFBP4
CSE1L	COL11A1	PMP22
CXADR	COL12A1	COL14A1
CXCR4	COL1A1	F13A1
CYFIP2	COL27A1	CD74
CYGB	COL3A1	HLA-DRA
DACH1	COL4A1	CSDA
DAPK1	COL4A2	TMEM47
DBH	COL5A1	SEMA3C
DCX	COL5A2	CCND2
DDC	COL6A1	MAFF
DDX39A	COL6A2	HLA-DRB4
DIABLO	COL6A3	FOXO1
DKK1	COPA	IGFBP4
DLK1	CPED1	ITPR3
DNAJB1	CPS1	SEMA3C
DNAJC6	CRABP2	MEOX2
DNAJC9	CREB3L2	BHLHB2
DNER	CREG1	PYGL
DPYSL2	CRELD2	ITPR3
DPYSL3	CRISPLD1	NGFR
DPYSL5	CRTAP	ZFP36
DTD1	CSRP1	HLA-DPA1

DUSP4	CTDSP2	SERPING1
EEF1A2	CTNNA1	PTPRZ1
EIF1B	CTSB	ZEB2
ELAVL2	CTSC	IFI16
ELAVL3	CTSO	CEBPB
ELAVL4	CXCL12	GNG12
EML4	CYBRD1	EMP1
EML6	CYFIP1	CD59
ENDOG	CYP26A1	IGFBP6
ENO2	CYR61	GPC3
EPB41L4A-AS1	DCAF6	TGFBI
ESRRG	DDOST	IFITM3
EVL	DDR2	FOS
EXOC5	DESI2	DHRS3
EYA1	DKK3	CFH
FABP6	DLC1	NR4A1
FAM107B	DLX1	COL9A3
FAM155A	DLX2	KLF9
FAM163A	DMD	EGR2
FAM167A	DNAJC1	ITGA6
FAM169A	DNAJC10	CXCL12
FAM171B	DNAJC3	ATP1A2
FAM60A	DNM3OS	IFITM1
FAXC	DPY19L1	FBLN5
FBLL1	DSE	HLA-DMA
FBXO8	DUSP14	ALDH1A3
FEV	DUSP5	EMP2
FHOD3	DUSP6	IFITM2
FIGNL1	EDEM1	CCL2
FKBP1B	EDNRA	NRXN1
FKBP4	EFEMP2	FOS
FOXM1	EGFR	LPL
FOXO3	EGR1	PON2
FSD1	EGR3	ANXA2
FZD3	EHD2	RNASE1
GABRB3	ELAVL1	MAL
GAL	ELF1	CREB3L2
GAP43	ELK3	PTPN13
GATA2	ELK4	ARHGEF10
GATA3	EMILIN1	VIM
GCH1	EMP1	COL15A1
GDAP1	ENAH	RCAN1
GDAP1L1	EPHA3	CSRP1
GDI1	EPS8	CD9
GDPD1	ERBIN	CCL2
GGCT	ERLIN1	NNMT
GGH	ERRFI1	RDX
GLCCI1	ETS1	IL6
GLDC	EVA1A	ANXA2
GLRX	EXT1	CTGF

GMNN	EXTL2	HLA-DQB1
GNB1	F2R	HLA-DQB1
GNG4	F2RL2	AXL
GPR22	FAM102B	RPESP
GPR27	FAM114A1	HSPA12A
GRB10	FAM120A	HLA-E
GRIA2	FAM129A	ANK3
H1FX	FAM3C	SRGN
HAND1	FAM43A	OLFML2A
HAND2-AS1	FAM46A	ITPR1
HES6	FAT1	GULP1
HEY1	FBN1	HRASLS3
HK2	FBN2	DAG1
HMGA1	FGFR1	LMNA
HMP19	FIBIN	SEPP1
HN1	FILIP1L	COL16A1
HNRNPA0	FKBP14	C10orf116
HS6ST2	FLNA	CNN3
ICA1	FLRT2	FER1L3
IGFBPL1	FMOD	PTGDS
IGSF3	FN1	RARRES2
INA	FNDC3B	LITAF
INO80C	FSTL1	LTBP4
INSM1	FUCA2	PDZD2
INSM2	FZD1	EHBP1
IRS2	FZD2	CD59
ISL1	FZD7	RNASE4
KDM1A	GABRR1	TM4SF1
KIAA1211	GALNT10	SYPL1
KIDINS220	GAS1	LAMA4
KIF15	GAS2	ERBB3
KIF1A	GDF15	MPZ
KIF21A	GJA1	ANGPTL7
KIF2A	GNAI1	SAMHD1
KIF5C	GNG12	MAF
KLC1	GNS	KLF6
KLF13	GORAB	ADH1B
KLF7	GPC6	IGFBP5
KLHL13	GPR137B	P2RY14
KLHL23	GPX8	PLEKHC1
KNSTRN	GRN	PTGDS
L1CAM	GSN	SPRY2
LEPROTL1	HES1	PTGDS
LIN28B	HEXB	THBS4
LINC00888	HIBADH	SERPINA3
LMO3	HIPK3	PLAT
LOC100507194	HIST1H2AC	ITGA7
LOC101928409	HIST1H2BK	C4A
LRRTM2	HLA-A	MT1E
LSM3	HLA-B	MAP4

LSM4	HLA-C	CLU
LYN	HLA-F	ITGA6
MAGI3	HLX	KIAA0256
MANEAL	HNMT	LRIG1
MAP1B	HOMER1	PTRF
MAP2	HS3ST3A1	NID1
MAP6	HSP90B1	CRLF1
MAPK8	HSPA5	CAV1
MAPT	HSPB1	MAP4
MARCH11	HTRA1	ID3
MCM2	HYOU1	DUSP6
MCM6	ID1	ARID5B
MCM7	ID3	PLS3
MIAT	IFI16	S100B
MMD	IFITM2	LAMC1
MRPL48	IFITM3	CASP4
MSH6	IGF2R	NPC2
MSI2	IGFBP5	AP1S2
MTCL1	IGFBP6	ABLIM3
MXI1	IL13RA1	FAM114A1
MYBL2	IL6ST	HSPG2
MYEF2	INSIG1	CX3CR1
MYO5A	IQGAP2	CFI
MYRIP	ITGA10	PDPN
NANOS1	ITGA4	PDLIM4
NAP1L5	ITGAV	LAMB1
NAPB	ITGB1	CTSC
NARS2	ITM2B	NDRG1
NBEA	ITM2C	ATF3
NCAM1	ITPR1	BCL6
NCAN	ITPRIPL2	PDGFRL
NCOA7	JAK1	CBX7
NCS1	JAM3	DARC
NEFL	KANK2	PRSS23
NEFM	KCNK2	IGFBP5
NELFCD	KCTD12	LAMP2
NELL2	KDELC2	ANXA2
NET1	KDELR2	EMP3
NFIL3	KDELR3	LITAF
NGRN	KDM5B	ANXA5
NMNAT2	KIAA1462	TACC1
NNAT	KIF13A	ITM2A
NOL4	KIRREL	PDGFA
NPTX2	KLF10	NR4A2
NPY	KLF4	S100A11
NRCAM	KLF6	AMOTL2
NRSN1	L3HYPDH	MDFIC
NSG1	LAMB1	BACH1
NUDT11	LAMC1	PTX3
NUF2	LAMP1	FCGR2A

NUSAP1	LAPTM4A	RGS1
OLA1	LASP1	ARHGEF6
OLFM1	LATS2	UGT8
PARP6	LEPROT	IL6ST
PBK	LGALS1	FEZ1
PBX3	LHFP	FOS
PDK1	LHX8	ITPR1
PEG3	LIFR	FGL2
PHF21B	LIPA	CFH
PHOX2A	LITAF	MAP4
PHOX2B	LIX1L	MOXD1
PHPT1	LMAN1	DMN
PHYHIPL	LMNA	NID2
PIK3R1	LOXL2	NR4A1
PKIA	LPP	LOC441019
PLPPR5	LRP10	AQP1
PNMA2	LRRC17	CRIM1
POLB	LRRC8C	FSTL3
POPDC3	LTBP1	EPS8
PPM1E	LUZP1	TM4SF1
PPP1R9A	MAGT1	FMO3
PPP2R3C	MAML2	TLN1
PRC1	MAN2A1	BIRC3
PRCD	MANF	IFITM1
PRIM1	MBD2	DUSP1
PRPH	MBNL1	BCAS1
PRSS12	MBTPS1	SRPX
PRSS3	MEOX1	TIMP3
PTS	MEOX2	CD99
QDPR	MEST	HLA-A
RAB33A	MGAT2	THBS2
RAB6B	MGP	PLSCR1
RALGDS	MGST1	RHOC
RANBP1	MICAL2	NFE2L2
RBBP8	MMP2	TYROBP
RBMS3	MOB1A	TSPAN8
RBP1	MRC2	MEGF9
REC8	MXRA5	KLC1
REEP1	MYADM	CTNNA1
RET	MYDGF	ZFP36L2
RFC4	MYL12A	TNFRSF1A
RGS17	MYL12B	MBNL1
RGS5	MYLIP	ARPC1B
RIMBP2	NANS	CAV2
RIMS3	NBR1	MYL9
RNF144A	NEK7	B2M
RNF150	NES	RAB13
RNF157	NFIA	ZFP36L2
RNF165	NFIC	DDIT4
RNFT2	NID1	JUNB

RPS6KA2	NID2	ABCA6
RRM2	NOTCH2	RPGR
RTN1	NOTCH2NL	IGF1
RTN2	NPC2	SLCO2B1
RUFY3	NPTN	PPAP2A
RUNDC3A	NQO1	TFPI
RUNDC3B	NR3C1	SPARC
SATB1	NRP1	INHBB
SBK1	OGFRL1	NEK7
SCAMP5	OLFML2A	CTNNA1
SCG2	OLFML2B	THBD
SCG3	OLFML3	RAB31
SCN3A	OSTC	AIF1
SEC11C	P4HA1	HTRA1
SEPT3	PALLD	VAMP5
SEPT6	PAPSS2	ADD3
SERP2	PCDH18	METTL7A
SETD7	PCOLCE2	MBD2
SHC3	PCSK5	CAST
SHD	PDE3A	ITGAV
SIX3	PDE7B	RRAS
SLC10A4	PDGFC	MT1F
SLC35G2	PDIA3	RARRES3
SLIT1	PDIA4	ADD3
SLIT3	PDIA6	MT1A
SNAP25	PDLIM1	HLA-B
SNAP91	PEA15	ABCA6
SOX11	PEAK1	LAMP2
ST3GAL6	PHLDA3	SH3BGRL
STMN2	PHLDB2	PLK2
STMN4	PHTF2	SGCE
STRA6	PIAS3	CTNNA1
STXBP1	PLAGL1	MCL1
SV2C	PLEKHA2	FCGBP
SYNPO2	PLEKHH2	NMI
SYT1	PLK2	HLA-DRB1
SYT4	PLOD2	MT1H
TACC2	PLOD3	RGL1
TAGLN3	PLPP1	RARRES1
TBC1D30	PLS3	COX7A1
TBPL1	PLSCR1	NEFH
TCEAL7	PLSCR4	TIMP3
TDG	PLXDC2	CTNNA1
TENM4	POLR2L	HLA-DMB
TFAP2B	PON2	STOM
TH	POSTN	RDX
THSD7A	PPIB	ZFP36L1
TIAM1	PPIC	CCL14
TMEM108	PPT1	IGFBP7
TMEM178B	PRCP	TLE1

TMEM97
TMOD1
TMOD2
TMTC4
TOX2
TRAP1
TSPAN13
TSPAN7
TTC8
TUB
TUBB2A
TUBB2B
TUBB3
TUBB4B
UBE2C
UBE2T
UCP2
UNC79
VRK1
ZNF195
ZNF22
ZNF24
ZNF512
ZNF536
ZNF704
ZNF711
ZNF738
ZNF91
ZWILCH

PRDM6
PRDX4
PRDX6
PROM1
PRRX1
PTBP1
PTGER4
PTGFRN
PTN
PTPN14
PTPRG
PTPRK
PTRF
PXDC1
PXDN
PYGL
QKI
QSOX1
RAB13
RAB29
RAB31
RAP1A
RAP1B
RBMS1
RCN1
RECK
REST
RGL1
RGS10
RGS3
RHOC
RHOJ
RIN2
RIT1
RNFT1
RNH1
ROBO1
ROR1
RRBP1
S1PR3
SASH1
SCPEP1
SCRG1
SDC2
SDC4
SDCBP
SDF4
SEC14L1
SEL1L3
SEMA3C

STAB1
MT1F
TMEM123
PTRF
MCL1
ANXA7
COL8A1
MAP3K7IP2
TXNIP
FILIP1L
EVI5
KIAA0494
NFKBIA
MSN
TOR1AIP1
THBS2
S100A4
DYNLT3
TGIF1
PLTP
HLA-F
EDNRB
ANXA11
MT3
TPSAB1
MT1B
DAAM2
SERTAD2
PDPN
EGFR
MYC
B2M
RRAS
SYNPO
SH3BGR
C21orf25
CLEC2B
ANXA2P1
GBP2
CD63
CREM
CTNNA1
DPYD
LOC731682
DAB2
PDE8A
CD58
FOSB
CH25H
KCNS3

SEMA3F	SKAP2
SEPT10	EDG2
SERPINE2	ERBB2
SERPINH1	ERBB2
SFT2D1	CAPN6
SFT2D2	CAT
SGK1	C10orf10
SH3BGRL	C1QB
SHC1	TNFRSF1B
SHROOM3	CREM
SIX1	JUNB
SIX4	MAN2A2
SKIL	RXRG
SLC16A4	SP100
SLC30A1	TPP1
SLC30A7	FBN1
SLC35F5	GNG11
SLC38A2	NR3C1
SLC38A6	IL15
SLC39A14	RASSF2
SMAD3	TSC22D3
SNAI2	CD14
SNAP23	IFIT3
SOSTDC1	VAMP3
SOX9	DUSP5
SPARC	FAT
SPARCL1	PLLP
SPATA20	TAX1BP3
SPCS3	IQGAP1
SPRED1	PLCE1
SPRY1	SLC2A3
SPRY4	HBEGF
SPRY4-IT1	TRAF1
SQSTM1	GPC1
SRPX	PPP1R15A
SSBP4	FCGRT
SSR1	PALLD
SSR3	MT1X
STAT1	CALD1
STAT3	ITGB8
STEAP1	LEPROT
STK38L	VCL
SUCLG2	RNASE6
SURF4	SAT1
SVIL	ADH1A
SYDE1	MAP4
SYNJ2	CD81
SYPL1	SASH1
TCF7L2	S100A13
TFE3	BTN3A3

TFPI
TGFB1I1
TGFBR2
THBS1
TIMP1
TJP1
TM4SF1
TM9SF2
TMBIM4
TMED9
TMEFF2
TMEM263
TMEM50A
TMEM87B
TNC
TNFRSF12A
TNFRSF1A
TNMD
TNS1
TOR1AIP1
TPBG
TPM1
TPM2
TRAM1
TRAM2
TRIL
TRIM5
TSC22D2
TSC22D3
TSPAN4
TUBB6
TWSG1
TXNDC12
UAP1
UGDH
VCL
VIM
WIPI1
WLS
WNT5A
WWTR1
YAP1
ZCCHC24
ZFP36L1
ZNF217

TNFAIP2
SLIT2
HSPB1
GPX3
GPR137B
C6orf145
FAM38A
NTRK2
TFAP2A
P4HA2
FLNA
HCLS1
SNCG
BTG2
LAMP1
TSPO
MEOX2
FCGRT
HLA-F
ANXA3
B2M
PLEC1
CALCOCO2
SDC4
FTH1
CXCL12
SPRY1
SERPINF1
LSP1
SSPN
TNFAIP6
TMED5
ZNF217
SCP2
BST2
GALC
TRAM2
CAPN2
GNAI2
TMED10
HTRA1
LAMB2
NFIL3
MRCL3
SELE
HEPH
PPP1R12B
TRIM22
PNRC1
PLXNB3

SSFA2
DOCK1
PEA15
ENG
LY96
CLEC3B
ID1
SNED1
LGI1
MVP
