# Supplementary material for: Tumor-suppressive disruption of cancer subtype-associated super enhancer circuits by small molecule treatment
Source: NAR Cancer. 2023 Feb 6;5(1):zcad007. doi: 10.1093/narcan/zcad007 (PMC9900422; doi:10.1093/narcan/zcad007)
Supplement: zcad007_Supplemental_Files [file zcad007_supplemental_files.zip › Koeniger_Suppl material methods_R2v1.pdf]

Koeniger et al. – Supplemental methods

Microscopy

Cells were seeded on etched cover slips and treated according to the respective experimental protocol. Subsequently, cells were fixed in 4% formaldehyde/PBS (10 min at RT), washed, and blocked with 10% serum/PBS for 1h at RT. Then, cover slips were incubated with the primary antibody in antibody-solution (PBS containing 10% serum and 0.1% Saponin) overnight at 4°C. After washing with PBS at RT, cover slips were incubated with fluorophore-coupled secondary antibodies diluted in antibody-solution at RT in the dark for 2 h. After washing with PBS and rinsing with water, cover slips were mounted with mounting medium containing DAPI (Vectashield). Confocal images were recorded using a confocal laser scanning microscope (Leica SP8i; Leica Microsystems, Wetzlar, Germany). For specific information an antibodies please see supplemental section.

Intracellular ALK staining

Cells were harvested, fixed in fixation buffer (Biolegend #420801) and permeabilized in Perm buffer (Biolegend #421002) according to manufacturer's instructions. After blocking in 1% FBS in PBS for 20 min, cells were stained with anti-ALK antibodies (Cell Signaling, #3633, dilution 1:400) or with normal rabbit IgG as a control (Santa Cruz, sc-2027, 1:400) followed by secondary anti-rabbit antibody conjugated to Alexa488 (Invitrogen, A-11070; 1:500) diluted in 1% FBS in PBS. The percentage of stained cells was measured by flow cytometry (Cytoflex, BeckmanCulter).

Western blotting

Separation of lysates by SDS-PAGE (Bio-Rad) and subsequent blotting on Immobilon-PVDF membranes (Millipore) was done as described in (Schneider et al., 2015), followed by incubation with the respective primary antibody. After incubation with a corresponding HRP-coupled secondary antibody the HRP signal was detected using Pierce ECL Western Blotting Substrate (Thermo Scientific) according to the manufacturer's protocol. For specific antibody information, please see supplement.

RNA/cDNA analysis

Total RNA was extracted using the NucleoSpin RNA II kit from Macherey-Nagel according to the manufacturer´s protocol. 1 µg of total RNA was used for cDNA synthesis using the iScript cDNA Synthesis Kit (BioRad). For quantitative PCR reactions the Absolute QPCR SYBR Green Mix (Thermo scientific) was used. qPCR reactions were performed on 96 well plates using either the Mx3000P or

Mx3005P qPCR systems (Agilent). Relative expression was calculated according to the $2^{\Delta\Delta Ct}$- method. For specific information on qPCR primer sequences please see supplement.

RNAseq

Cells (SH-SY5Y) were grown in DMEM (high glucose) with 0.5% FBS, treated with 20 µM ISX (or DMSO control) for 48 h hours and RNA was extracted using the Macherey-Nagel NucleoSpin RNA Isolation Kit, according to the manufacturer's protocol. Integrity of total RNA was assessed on the Bio-Rad Experion. RNAseq libraries were generated using Lexogen Quantseq, according to the manufacture's protocol and sequenced on an Illumina NextSeq 550 using 75 bp single end reads. Raw reads were aligned to the human genome (GRCh38) using STAR (Dobin et al., 2013) version 2.6.1d, and quantified as Counts per Million (CPM) against the protein- and lincRNA-coding genes as defined by Ensembl (Cunningham et al., 2022), revision 104.

Immunohistochemistry

For immunohistochemistry, heat-induced epitope retrieval was performed with EDTA. Staining was performed on a DAKO Autostainer-Plus. After blocking endogenous peroxidase, sections were incubated for 45 minutes with the corresponding primary antibody. Sections were washed and incubated with Dako REAL EnVision HRP Rabbit/Mouse polymer, which reacts with DAB-Chromogen, according to the manufacturer´s protocol.

Koeniger et al. – Supplemental material

qPCR primer sequences (human, 5'→3') for qRT-PCR:

Transcript	Primer sequence	
NPM1	hNPM1_qFor	CTGGAGGTGGTAGCAAGGTTCCACAGA
	hNPM1_qRev	TGGCGCTTTTTCTTCAGCTTCCTCA
ODC1	hODC1_qFor	ATGGCTTCCAGAGGCCGACGATCTA
	hODC1_qRev	CATCCCACTCTCCCAGGCACAAGAC
CCNA2	hCCNA2_qFor	CAAAGCACCACAGCATGCACAACAG
	hCCNA2_qRev	CTGGTGGGTTGAGGAGAGAAACACCA
VIM	hVIM_qFor	TACCGGAGACAGGTGCAGTCCCTCA
	hVIM_qRev	TCACGAAGGTGACGAGCCATTTCCT
PRRX1	hPRRX1_qFor	TCCGTCAGTCACCTGCTAGACCTGGA
	hPRRX1_qRev	GTCCGCTCAAAGACACGCTCCAAAG
SNAI2	hSNAI2_qFor	CCAGACCCTGGTTGCTTCAAGGACA
	hSNAI2_qRev	TGCTCTGTTGCAGTGAGGGCAAGAA
YAP	hYAP1_qFor	ACAGTGTCCCTCGAACCCCAGATGA
	hYAP1_qRev	CCAAGGTCCACATTTGTCCCAGGAA
DLK1	hDLK1_qFor	AGAAAAAGGACGGGCCCTGTGTGA
	hDLK1_qRev	CTGTTGGCCACGATCTCGCAGAAA
SATB1	hSATB1_qFor	CCCCACATTATCCATGTTCCAGCAGA
	hSATB1_qRev	CCGGGTCTTCTGTCGGTTTTCCTCA
ASCL1	hASCL1_qFor	GGAGCAGGAGCTTCTCGACTTCACCA
	hASCL1_qRev	CCTCCCAACGCCACTGACAAGAAAG
HAND1	hHAND1_qFor	AGGCTGAACTCAAGAAGGCGGATGG
	hHAND1_qRev	CAGCCGGTGCGTCCTTTAATCCTCT
ALK	hALK_qFor	TAGAAAGGGAGGCTGTGCCATGCTG
	hALK_qRev	TCCTGGTTGCTTTTGCTGGGGTATG
MYCN	hMYCN_qFor	GTCTTCCCCTTTCCCGTGAACAAGC
	hMYCN_qRev	CCACAGTGACCACGTCGATTTCTTCC
PHOX2A	hPHOX2A_qFor	CGCTGAGACCCACTACCCCGACATT
	hPHOX2A_qRev	GCGCTCCTGTTTGCGGAACTTG
PHOX2B	hPHOX2B_qFor	GCTCAAAGAGCTGGAAAGGGTCTTCG
	hPHOX2B_qRev	GCGCTCCTGCTTGCGAAACTTG
TGM2	hTGM2_qFor	TCCTCGTGGAGCCAGTTATCAACAGC
	hTGM2_qRev	ACCTCAGCCACCAGCTTGCGTTTCT
FN1	hFN1_qFor	CAGTGGAATGCACCACAGCCATCTC
	hFN1_qRev	TGGTAGCTTCCTTCCAACGGCCTACA
DKK3	hDKK3_qFor	GGCTTGACAGCATGAGGTGTTGTGC
	hDKK3_qRev	TAACCCTGCCTGACTCTCCCAAGCA
PLAUR	hPLAUR_qFor	CCGAGGCCCCATGAATCAATGTCT
	hPLAUR_qRev	GGCGGTTGCACAGCCTCTTACCATA
SMG1	hSMG1_qFor	GGGCCGAGATGTTGATCCGAATAGG
	hSMG1_qRev	CACCCAGGCTGTCCAACCTTCATACA
FABP5	hFABP5_qFor	CCACCATGGCCACAGTTCAGCA
	hFABP5_qRev	ATTGCGCCCATTTTTCGCAAAGCTA

UFD1L	hUFD1L_qFor<br>hUFD1L_qRev	TGGCTGATGAGGGCATCTGCTACCT<br>GCCACTTGAAGGTTGACGCTCTCCA
WWTR1	hTAZ_qFor<br>hTAZ_qRev	AGTGGCCTGGGGTTAGGGTGCTACA<br>CGGGTCTGTTGGGGATTGATGTTCA
CREG1	hCREG1_qFor<br>hCREG1_qRev	GGTGGACCAAAAATCGTGACACCAGA<br>CAAGCAAGCAAACAAACCAGCATGTGA
MEOX1	hMEOX1_qFor<br>hMEOX1_qRev	TGGGAGCACTGCCAATGAGACAGAG<br>CCCTTCACACGCTTCCACTTCATCC
MEOX2	hMEOX2_qFor<br>hMEOX2_qRev	GCAAACCCAGGAAAGAAAGGACAGCA<br>ACCCTCTTCCACTTCATCCGCCTGT
GATA2	hGATA2_qFor<br>hGATA2_qRev	GTCTGCAACGCCTGTGGCCTCTACT<br>CTCCGCCCCTTTCTTGCTCTTCTTG
GATA3	hGATA3_qFor<br>hGATA3_qRev	CTGTCTGCAATGCCTGTGGGCTCTA<br>CGGTTTCTGGTCTGGATGCCTTCCT
EYA1	hEYA1_qFor<br>hEYA1_qRev	CTGTGACCAGGGACAGATCCAGCAG<br>AGACTGACAGCAACTGCGCATCACC
DACH1	hDACH1_qFor<br>hDACH1_qRev	GCGGCAGAACAGATGCTGAAAGGAC<br>TCCCATGACGAATGTCTGACTGCAAA
p75NGFR	hP75_qFor<br>hP75_qRev	ACTCCACAGCGACAGTGGCATCTCC<br>GGCAGGCCTCATGGGTAAAGGAGTCTA
NGF	hNGF_qFor<br>hNGF_qRev	CTGCCCCCTTCAACAGGACTCACAG<br>TCTTATCCCCAACCCACACGCTGAC
BDNF	hBDNF_qFor<br>hBDNF_qRev	GGAAGAGGCCAGGACAGAGCTGACA<br>TCCATGAACAGACAGGATGGGCAGA
NT3 (=NTF3)	hNT3_qFor<br>hNT3_qRev	GCCACACGGATGCCATGGTTACTTT<br>TTGGATGCCACGGAGATAAGCGAGA
NT4	hNT4_qFor<br>hNT4_qRev	GGTGCTGACAGGTGCTCCGAGAGAT<br>GCACACTGGGGAGGAGGAAAAGGAG
GDNF	hGDNF_qFor<br>hGDNF_qRev	TGAGACCAGCTTGGGCAACATAGGG<br>CAGCTCACTGCAGCCTCGACCTTCT
CNTF	hCNTF_qFor<br>hCNTF_qRev	GTCGGGACCTCTGTAGCCGCTCTATCT<br>CCATCCGCAGAGTCCAGGTTGATGT
RPLP0	hP0_qFor<br>hP0_qRev	CCTTCTCCTTTGGGCTGGTCATCCA<br>CAGACACTGGCAACATTGCGGACAC

qPCR primer sequences (human, 5'→3') for ChIP-PCR:

Genomic locus	Primer sequence	
***MEIS1* (SE)**	For:	GGGACTGTTGACTTTGTC
	Rev:	GAAGTCTCCAGGCTACTAC
***ODC1* (intron 1)**	For:	AGCCCCGAGCTGTGAAGA
	Rev:	GCGGCTGGATAAGGGTGA
***FASN* (promoter)**	For:	GAGGGAGCCAGAGAGACGGC
	Rev:	CCGGCTGCTCGTACCTGG

Antibodies

Target	Company	Catalogue details	Application
ALK	Cell Signaling Technology	#3633	WB, Flow
N-MYC	Cell Signaling Technology	#9405	WB
β-Actin	Sigma	A5441	WB
P75NGFR	Cell Signaling Technology	#8238	IF, WB
SNAI2	Cell Signaling Technology	#9585	WB
NOTCH3	Cell Signaling Technology	#5276	WB
DBH	Cell Signaling Technology	#8586	WB
α-Tubulin	Sigma	T6199	WB
H3K27ac	Cell Signaling Technology	#8173	IF, WB
N-MYC	Cell Signaling Technology	#84406	ChIP
IgG pool	Sigma	I5006	ChIP
panH4ac (K5, K8, K12, K16)	Millipore	#06-866	ChIP
H3K27ac	Diagenode	C15410174	ChIP
Rabbit IgG	Santa Cruz	sc-2027	Flow
SYP	Cell Signaling Technology	#36406	IHC, IF
Ki67	Cell Signaling Technology	#9449	IHC
