# Supplementary material for: Tumor-suppressive disruption of cancer subtype-associated super enhancer circuits by small molecule treatment
Source: NAR Cancer. 2023 Feb 6;5(1):zcad007. doi: 10.1093/narcan/zcad007 (PMC9900422; doi:10.1093/narcan/zcad007)
Supplement: zcad007_Supplemental_Files [file zcad007_supplemental_files.zip › SupplFig_legends_R2v1.pdf]

Koeniger et al.

Supplemental figure legends

Figure S1

- **(A)** Changes in *ALK* mRNA levels upon DOX-mediated induction of *PRRX1A* expression in SKNBE(2)-C cells, mediating an ADRN-to-MES transition (van Groningen *et al.*, 2017).
- **(B)** Changes in *PHOX2A* mRNA (ADRN marker) levels upon DOX-mediated induction of *PRRX1A* expression in SKNBE(2)-C cells, mediating an ADRN-to-MES transition (van Groningen *et al.*, 2017).
- **(C)** Changes in *MYCN* mRNA levels upon DOX-mediated induction of *PRRX1A* expression in SKNBE(2)-C cells, mediating an ADRN-to-MES transition (van Groningen *et al.*, 2017).

Figure S2

- **(A)** Flow cytometry-based determination of ALK-positive cells in DMSO/ISX-treated SH-SY5Y cells. ALK-positive cells are colored in red, negative cells in green. Drug treatment (20 µM ISX) was for 48 h.
- **(B)** Quantification of three parallel experiments as outlined in (A).
- **(C)** N-MYC levels (Western blot) in IMR32 cells treated with the indicated concentrations of ISX (48 h treatment duration). Shown is one experiment of n=2.
- **(D)** N-MYC levels (Western blot) in IMR32 cells treated with 20 µM ISX for the indicated time periods. Shown is one experiment of n=2.
- **(E)** Spike-in-normalized ChIPseq track of N-MYC binding at the *NCL* promoter (MYC target gene) in IMR32 cells exposed to DMSO or ISX (20 µM) for 8h.
- **(F)** Anti N-MYC ChIP-qPCR of IMR32 cells treated with DMSO or ISX (20 µM) for 8h. Shown is one experiment measured in triplicate (mean ±SD).
- **(G)** Transcriptomic cell cycle-related signatures repressed by ISX (20 µM, 48h) in IMR32 cells (RNAseq, downregulated genes, cut-off 1.5-fold).
- **(H)** Chromatin-related transcriptional signatures (RNAseq, upregulated genes, cut-off 2-fold) from IMR32 cells treated with ISX (20 µM) for 48h. DMSO-treated cells served as control.

Figure S3

- **(A)** Relative mRNA expression (qPCR) of ADRN/MES marker genes in SKNBE(2) cells treated with DMSO or ISX (20 µM) for 48 h. Shown is the mean of n≥3 ±SD.
- **(B)** Relative mRNA expression (qPCR) of ADRN/MES marker genes in GIMEN cells treated with DMSO or ISX (20 µM) for 48 h. Shown is the mean of n≥3 ±SD.
- **(C)** Graphical representation of up/down-regulated MES signature genes upon ISX (20 µM, 48 h) treatment of IMR32 cells cut-off 2-fold (upregulated genes)/1.5-fold (downregulated genes)). Numbers indicate the number of genes regulated. P value determined by hypergeometric test.
- **(D)** Graphical representation of up/down-regulated ADRN signature genes upon ISX (20 µM, 48 h) treatment of IMR32 cells cut-off 2-fold (upregulated genes)/1.5-fold (downregulated genes)). Numbers indicate the number of genes regulated. P value determined by hypergeometric test.
- **(E)** Time-dependent downregulation of ADRN marker gene expression (*HAND1, DBH*) in ISX (20 µM)-treated IMR32 cells. Shown are mean values of triplicate qPCR measurements of one experiment of total n=2.
- **(F)** Time-dependent upregulation of MES marker gene expression (*PRRX1, MEOX2*) in ISX (20 µM)-treated IMR32 cells. Shown are mean values of triplicate qPCR measurements of one experiment of total n=2.
- **(G)** MA plot depicting all transcripts (RNAseq) altered upon ISX treatment (IMR32 cells, 20µM iSX for 48 h). MES and ADRN signature genes are colored in green and red, respectively.
- **(H)** PCA plot (RNAseq) from IMR32 and SH-SY5Y cells exposed to DMSO or ISX (20 µM for 48 h). For comparison, ADRN (red), MES (green) and NC (gray) samples from (van Groningen *et al.*, 2017) have been included. Note that ISX induces a shift from ADRN towards MES/NC.
- **(I)** Heatmap depicting the top 500 ISX-induced genes (IMR32 cells, 20µM ISX, 48h) during ADRN-to-MES transition upon DOX-induced *PRRX1* expression in SKNBE(2)-C cells (van Groningen *et al.*, 2017).

Figure S4

(A) Relative mRNA expression changes (qPCR) of the indicated neurotrophins and *p75NGFR* in ISX (20 µM; 48h)-treated SH-SY5Y cells. Shown is the mean of n=3 ±SD.

(B) Western blot depicting protein abundance changes of p75NTR upon ISX treatment (20 µM; 72h). Shown is one experiment of n=2.

(C) Confocal images of non-permeabilized SH-SY5Y cells treated with DMSO or 20 µM ISX for 48 h. p75NTR: Green; Nuclei: Blue (DAPI). Scale bar 10 µm. The inset shows a magnification to demonstrate membranous localization of p75.

(D) *NGFR (=p75NTR; =p75)* mRNA expression in a cellular model of MES/ADRN/NC differentiation (van Groningen *et al.*, 2017). MES (mesenchymal; n=7); ADRN (adrenergic; n=22); NC (neural crest; n=5). Shown is the mean ±SD.

(E) *NGF* mRNA expression in a cellular model of MES/ADRN/NC differentiation (van Groningen *et al.*, 2017). MES (mesenchymal; n=7); ADRN (adrenergic; n=22); NC (neural crest; n=5). Shown is the mean ±SD.

(F) *NT3 (=NTF3)* mRNA expression in a cellular model of MES/ADRN/NC differentiation (van Groningen *et al.*, 2017). MES (mesenchymal; n=7); ADRN (adrenergic; n=22); NC (neural crest; n=5). Shown is the mean ±SD.

(G) *GLI1* mRNA expression in a cellular model of MES/ADRN/NC differentiation (van Groningen *et al.*, 2017). MES (mesenchymal; n=7); ADRN (adrenergic; n=22); NC (neural crest; n=5). Shown is the mean ±SD.

(H) Graphical representation of up/down-regulated GN signature genes upon ISX (20 µM, 48 h) treatment of IMR32 cells (RNAseq, cut-off 2-fold (upregulated genes)/1.5-fold (downregulated genes)). Numbers indicate the number of genes regulated. P value determined by hypergeometric test.

(I) Overall survival (SEQC cohort) in relationship to MES score. Median split.

Figure S5

(A) K-means clustering of NB patient transcriptomes into three subgroups (SEQC cohort; 498 patients; (SEQC/MAQC III-Consortium, 2014)).

(B) Principle component analysis (PCA) of the three clusters determined in (A).

(C) INSS stage distribution among the three transcriptional subgroups identified in (A). Each small circle represents one patient.

(D) Kaplan-Meier plot depicting overall survival of the three patient groups identified in (A).

(E) Heatmap depicting ISX-induced genes (top 500, IMR32, 48h of ISX (20µM) treatment) within the SEQC patient cohort (grouped according to k-means clustering). Note that ISX-induced transcripts are enriched in group 2 patients.

(F) Heatmap depicting ISX-induced genes (top 500, IMR32, 48h of ISX (20µM) treatment) within the Kocak (Kocak *et al.*, 2013) patient cohort (grouped according to k-means clustering). Note that ISX-induced transcripts are enriched in group 2 patients.

Figure S6

(A) Time-dependent reduction of cellular HDAC (classI/II) activity in IMR32 cells upon treatment with DMSO, ISX (20 µM) or SAHA (pan-HDAC inhibitor, positive control, 1 µM). Shown is one experiment (of n=2) measured in triplicate (mean ±SD).

(B) Anti-H3K27ac ChIP-qPCR (H3K27 acetylation at the *MEIS1* SE) of IMR32 cells treated with DMSO or ISX (20 µM) for 8h. Shown is one experiment measured in triplicate (mean ±SD).

(C) Spike-in-normalized ChIPseq track of N-MYC binding at the *MEIS1* super enhancer region in IMR32 cells exposed to DMSO or ISX (20 µM) for 8h.

(D) Spike-in-normalized ChIPseq track of N-MYC binding at the *MYCN* super enhancer region in IMR32 cells exposed to DMSO or ISX (20 µM) for 8h.

(E) Average plot depicting N-MYC binding at SEs (IMR32 +DMSO/ISX (20µM) for 8h; spike-in-normalized data). SE calling was done by ROSE.

(F) Average plot depicting H3K27 acetylation at normal enhancers (IMR32 +DMSO/ISX (20µM) for 8h; spike-in-normalized data).

Figure S7:

- **(A)** Summary of ATAC peaks and associated transcriptional changes.
- **(B)** Example of ATACseq result at the human *MYCN* gene locus (IMR32; ISX 20µM for 48h).
- **(C)** Average read plots comparing ATAC (48h ISX) signals with H3K27ac (8h ISX) changes in IMR32 cells. Only ATAC peaks gained by ISX are depicted (ATACup).
- **(D)** Principle component analysis (PCA) of ATACseq peaks (up+down) of two DMSO- and two ISX (20 µM, 48h)-treated IMR32 samples.
- **(E)** Transcriptomic terms associated with nearest genes of ISX-induced ATAC peaks (ATACup) in IMR32 cells.
- **(F)** ADRN/MES marker gene expression (qPCR) in xenograft tumor tissue at the end of the experiment. Each dot represents one tumor sample (mean ±SD).
- **(G)** Histological analysis of xenograft tumor tissue at the end of the experiment. HE=Hematoxylin/Eosin staining. SYP=Synaptophysin. Ki67=Ki67 proliferation marker. Scale bar is 50 µm.
- **(H)** Quantification of Ki67 labelling as shown in (F) (mean ±SD). Each dot represents one tumor.
- **(I)** *Synaptophysin* (*SYP*) mRNA expression in a cellular model of MES/ADRN/NC differentiation (van Groningen *et al.*, 2017). MES (mesenchymal; n=7); ADRN (adrenergic; n=22); NC (neural crest; n=5). Shown is the mean ±SEM.
- **(J)** Immunofluorescent staining of endogenous SYP (green) in DMSO- and ISX-treated (20 µM, 24 h treatment) IMR32 cells. Images are maximum intensity projections of z-stacks. Nuclei appear in blue (DAPI). Scale bars are 5 µm.
- **(K)** SYP staining intensity of cells depicted in (I). Shown is the fluorescence intensity per cell (mean ±SD). Each dot represents one cell.

Figure S8:

(A) Relative mRNA expression (array) of ADRN/MES marker genes in IMR32 cells treated with DMSO or THZ1 (CDK7i; 100nM). Shown is the change upon THZ1 treatment. Mean of n=3 ±SD. (Chipumuro *et al.*, 2014).

References

Chipumuro E, Marco E, Christensen CL, Kwiatkowski N, Zhang T, Hatheway CM *et al.* (2014). CDK7 inhibition suppresses super-enhancer-linked oncogenic transcription in MYCN-driven cancer. *Cell* **159**: 1126–1139.

Kocak H, Ackermann S, Hero B, Kahlert Y, Oberthuer A, Juraeva D *et al.* (2013). Hox-C9 activates the intrinsic pathway of apoptosis and is associated with spontaneous regression in neuroblastoma. *Cell death & disease* **4**: e586.

SEQC/MAQC III-Consortium. (2014). A comprehensive assessment of RNA-seq accuracy, reproducibility and information content by the Sequencing Quality Control Consortium. *Nature biotechnology* **32**: 903–914.

van Groningen T, Koster J, Valentijn LJ, Zwijnenburg DA, Akogul N, Hasselt NE *et al.* (2017). Neuroblastoma is composed of two super-enhancer-associated differentiation states. *Nature genetics* **49**: 1261–1266.
