# Supplementary figures and images for: Tumor-suppressive disruption of cancer subtype-associated super enhancer circuits by small molecule treatment
Source: NAR Cancer. 2023 Feb 6;5(1):zcad007. doi: 10.1093/narcan/zcad007 (PMC9900422; doi:10.1093/narcan/zcad007)

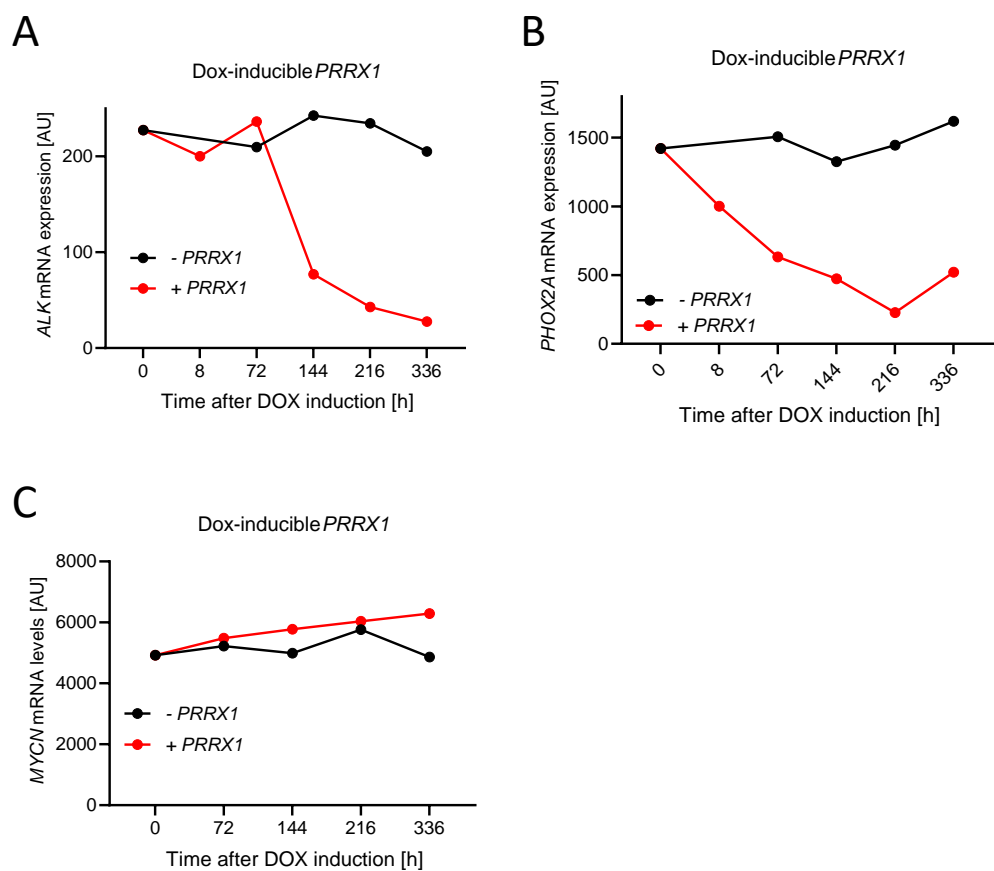

- Figure S1 -

Supplement: zcad007_Supplemental_Files [file zcad007_supplemental_files.zip › Fig S1.pdf]

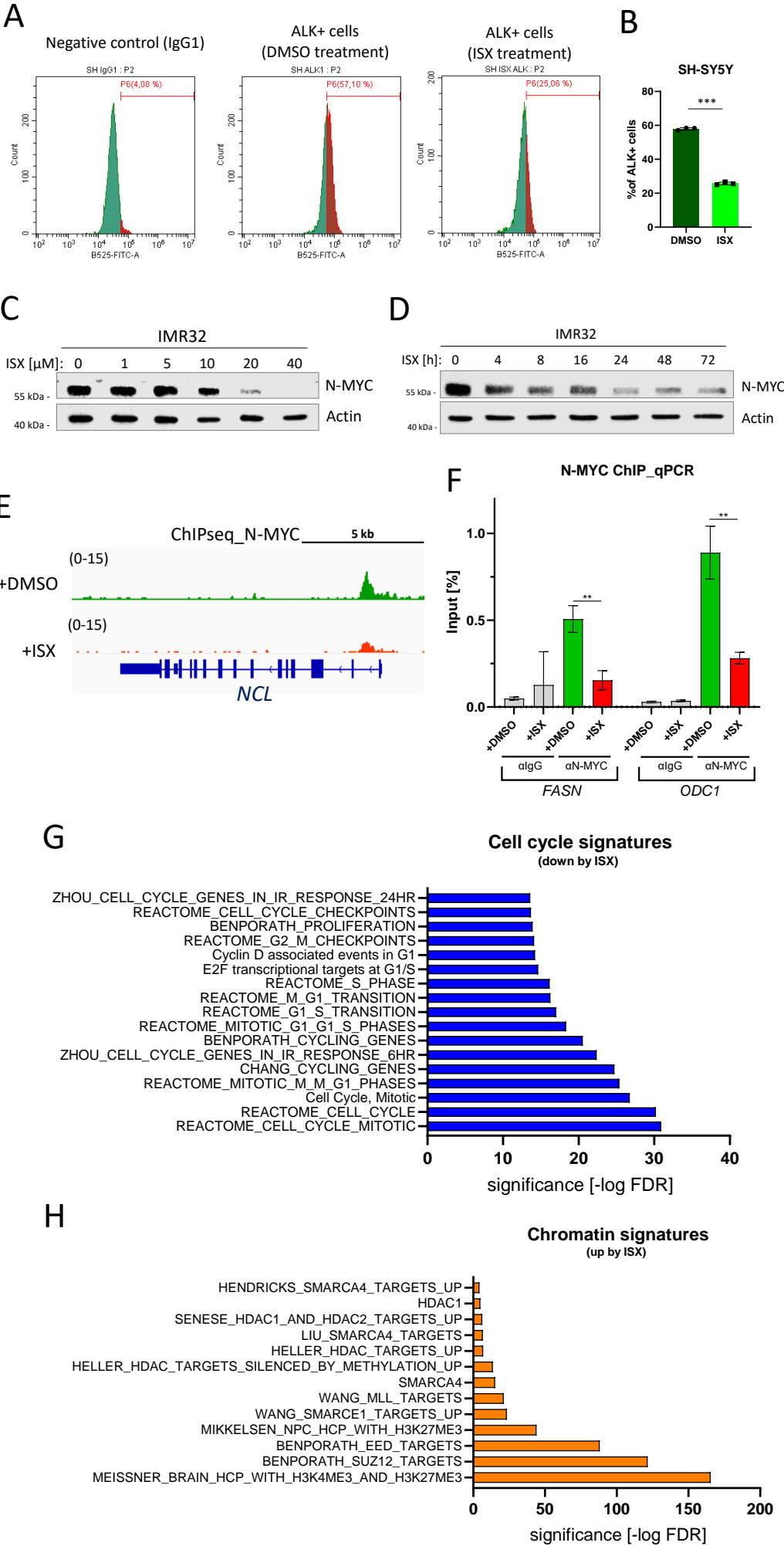

- Figure S2 -

Supplement: zcad007_Supplemental_Files [file zcad007_supplemental_files.zip › Fig S2.pdf]

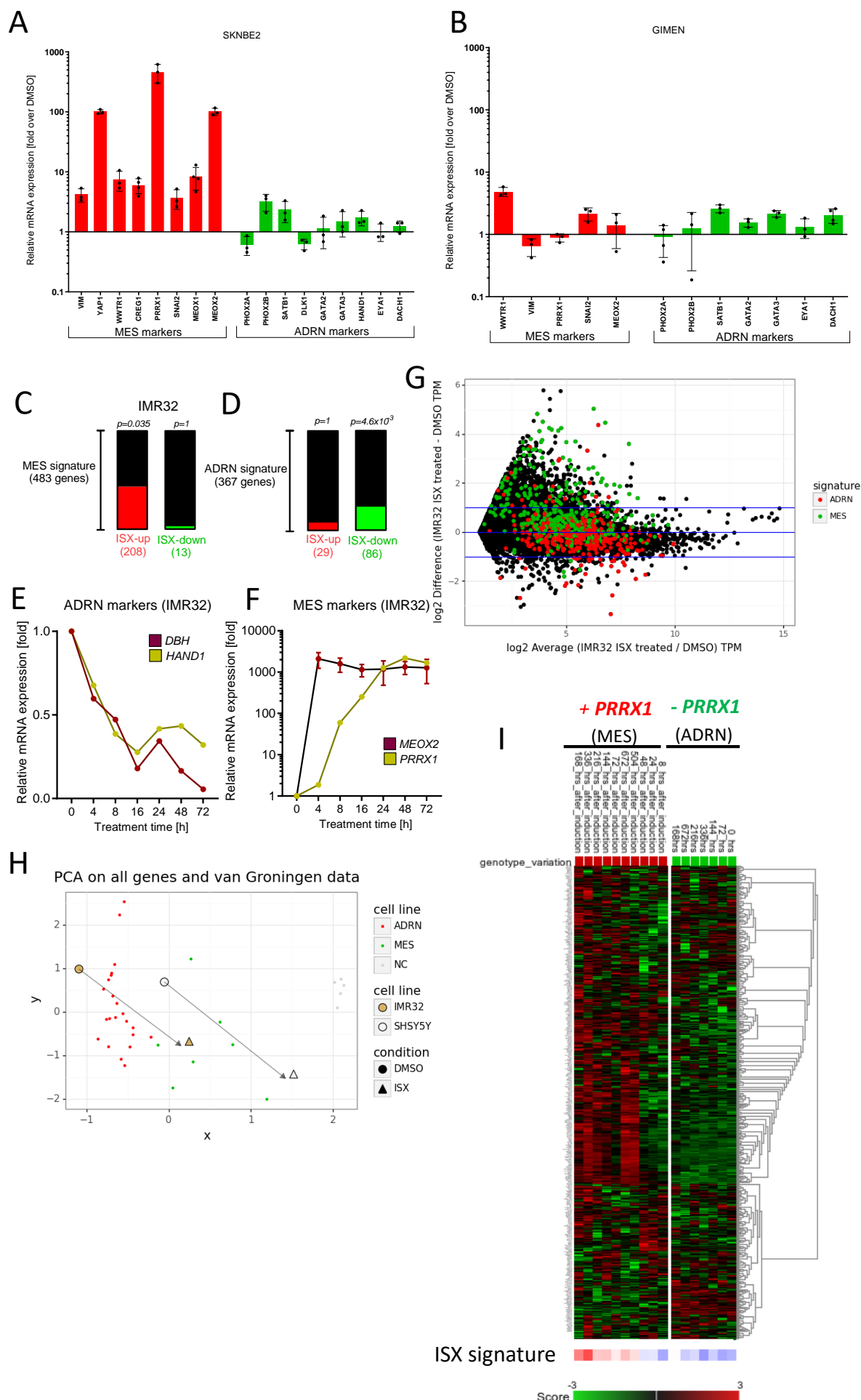

- Figure S3 -

Supplement: zcad007_Supplemental_Files [file zcad007_supplemental_files.zip › Fig S3.pdf]

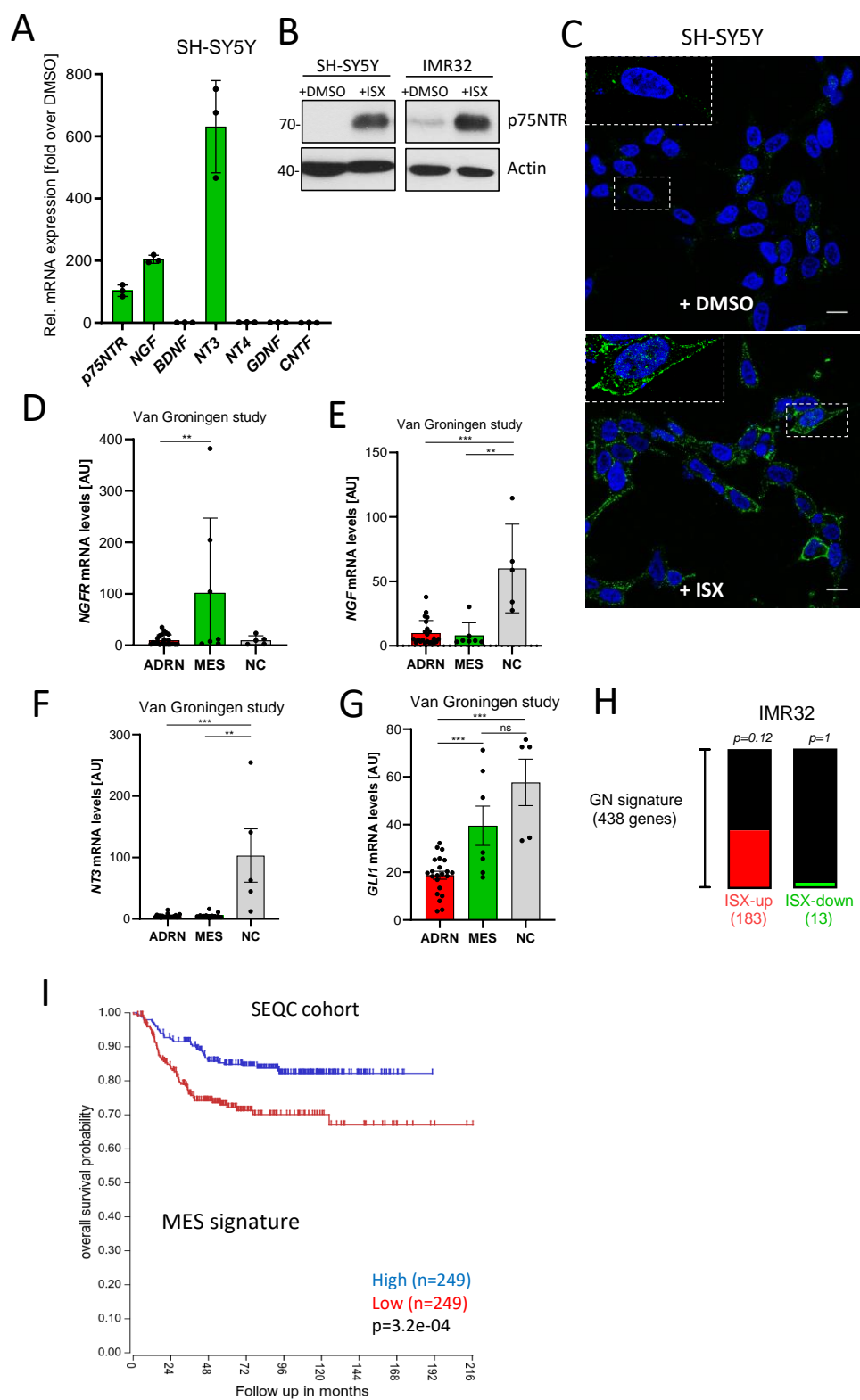

- Figure S4 -

Supplement: zcad007_Supplemental_Files [file zcad007_supplemental_files.zip › Fig S4.pdf]

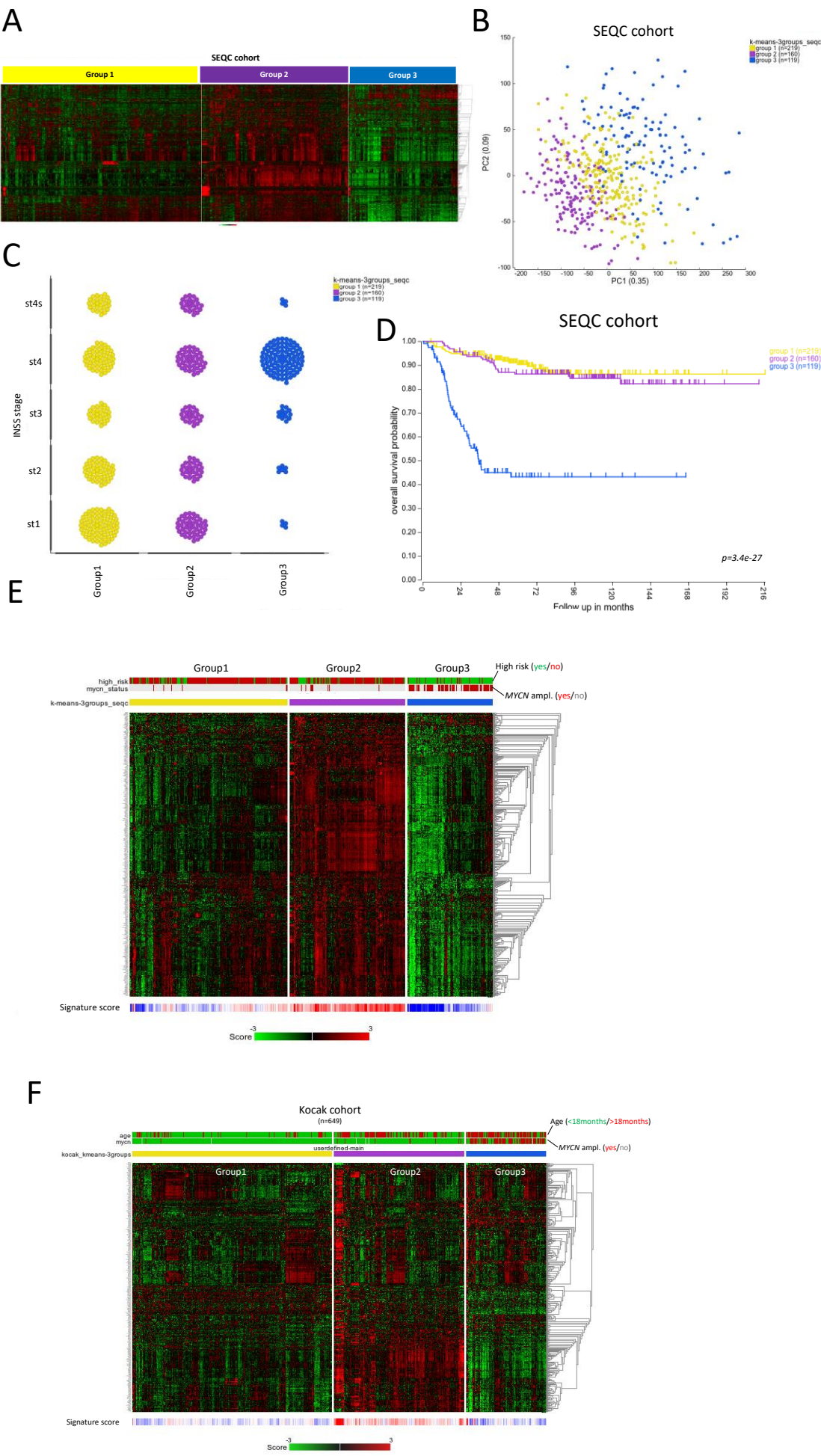

- Figure S5 -

Supplement: zcad007_Supplemental_Files [file zcad007_supplemental_files.zip › Fig S5.pdf]

A

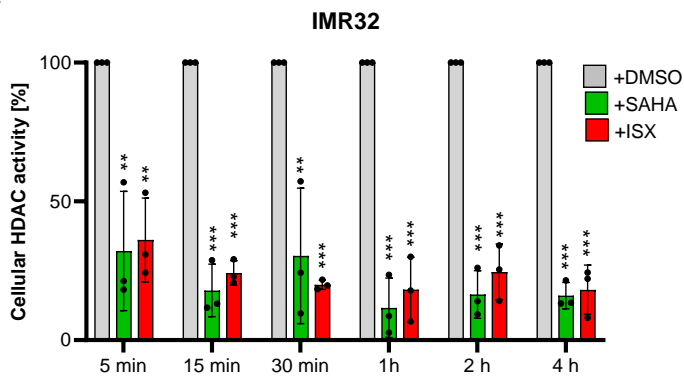

B

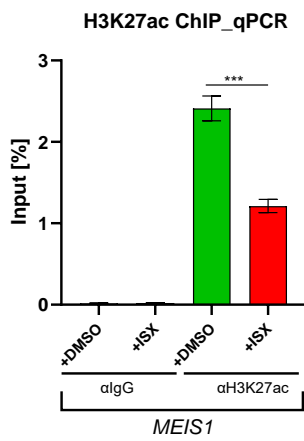

C

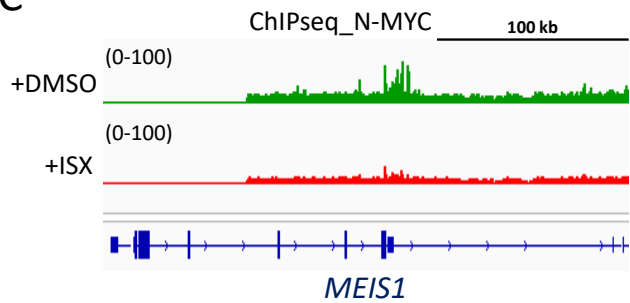

D

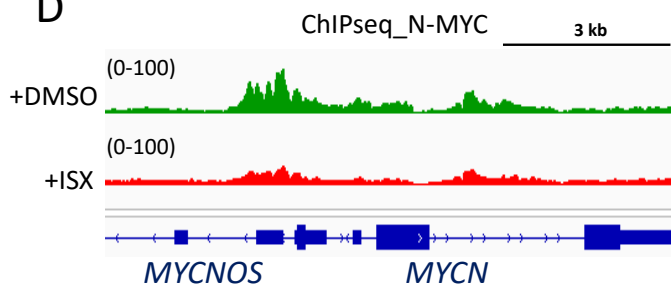

E

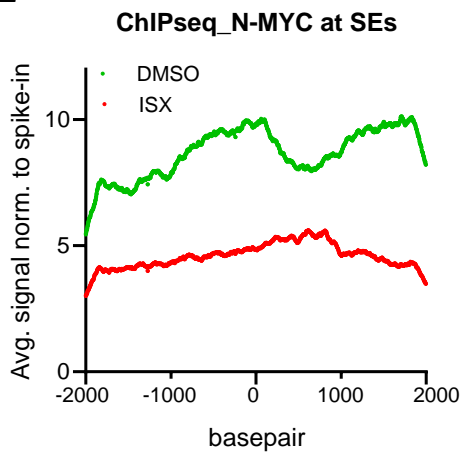

F

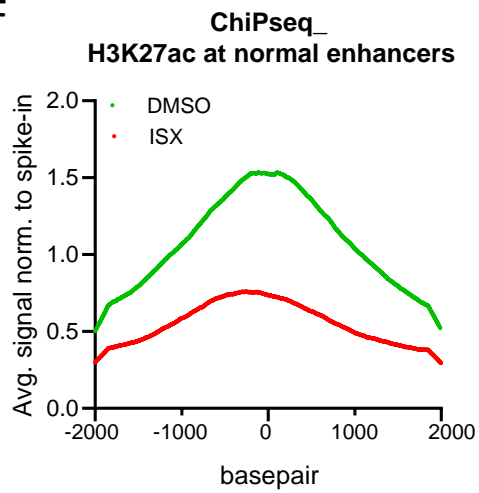

Supplement: zcad007_Supplemental_Files [file zcad007_supplemental_files.zip › Fig S6.pdf]

A

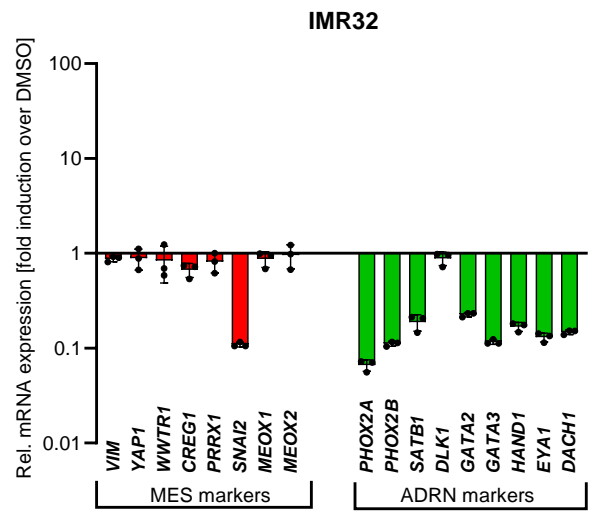

- Figure S8 -

Supplement: zcad007_Supplemental_Files [file zcad007_supplemental_files.zip › Fig S8.pdf]
