# Supplementary material for: Tumor-suppressive disruption of cancer subtype-associated super enhancer circuits by small molecule treatment
Source: NAR Cancer. 2023 Feb 6;5(1):zcad007. doi: 10.1093/narcan/zcad007 (PMC9900422; doi:10.1093/narcan/zcad007)
Supplement: zcad007_Supplemental_Files [file Fig S7.pdf › Fig S7.pdf]

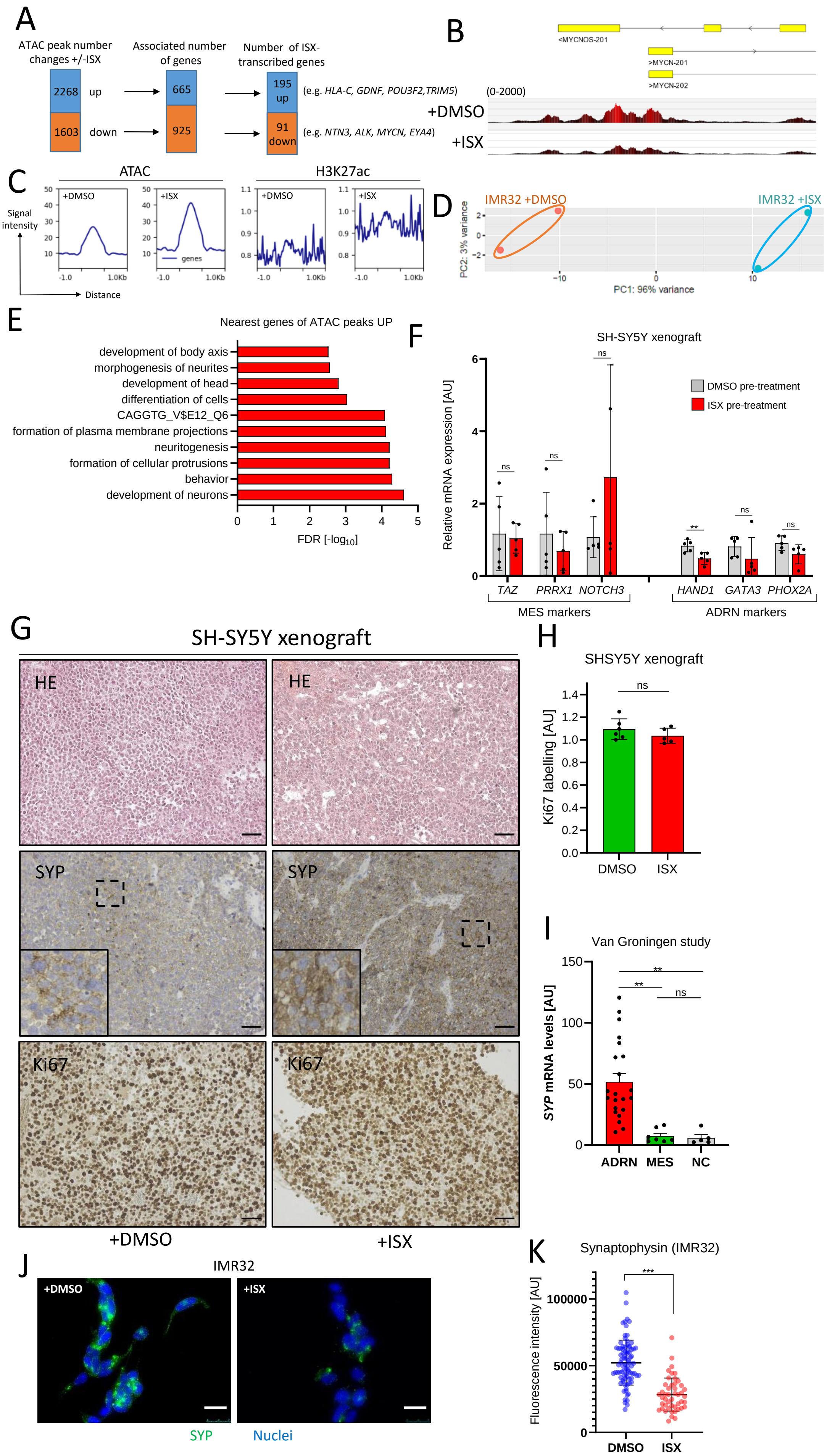
A
ATAC peak number changes +/-ISX
Associated number of genes
Number of ISX-transcribed genes
2268 up
1603 down
665
925
195 up
91 down
(e.g. HLA-C, GDNF, POU3F2,TRIM5)
(e.g. NTN3, ALK, MYCN, EYA4)
B
<MYCNOS-201
>MYCN-201
>MYCN-202
(0-2000)
+DMSO
+ISX
C
ATAC
H3K27ac
Signal intensity
Distance
+DMSO
+ISX
genes
D
IMR32 +DMSO
IMR32 +ISX
PC2: 3% variance
PC1: 96% variance
E
Nearest genes of ATAC peaks UP
development of body axis
morphogenesis of neurites
development of head
differentiation of cells
CAGGTG_V$E12_Q6
formation of plasma membrane projections
neuritogenesis
formation of cellular protrusions
behavior
development of neurons
FDR [-log10]
F
SH-SY5Y xenograft
DMSO pre-treatment
ISX pre-treatment
Relative mRNA expression [AU]
TAZ
PRRX1
NOTCH3
HAND1
GATA3
PHOX2A
MES markers
ADRN markers
G
SH-SY5Y xenograft
HE
SYP
Ki67
+DMSO
+ISX
H
SHSY5Y xenograft
Ki67 labelling [AU]
DMSO
ISX
I
Van Groningen study
SYP mRNA levels [AU]
ADRN
MES
NC
J
IMR32
+DMSO
+ISX
SYP
Nuclei
K
Synaptophysin (IMR32)
Fluorescence intensity [AU]
DMSO
ISX

- Figure S7 -
